# Regulation of coral assemblages: Spatial and temporal variation in the abundance of recruits, juveniles, and adults

**DOI:** 10.1371/journal.pone.0329546

**Published:** 2025-08-21

**Authors:** Radonirina Lebely Botosoamananto, Gildas Todinanahary, Lahitsiresy Max Gasimandova, Mahery Randrianarivo, Lucie Penin, Mehdi Adjeroud

**Affiliations:** 1 Institut Halieutique et des Sciences Marines (IH.SM), Université de Toliara, BP 141, Mahavatse II, Toliara, Madagascar; 2 UMR ENTROPIE - Faculté des Sciences et Technologies - Université de La Réunion & Laboratoire d’Excellence CORAIL - 15, avenue René Cassin - CS St Denis Cedex 9, La Réunion, France; 3 ENTROPIE, IRD, Université de la Réunion, CNRS, IFREMER, Université de la Nouvelle-Calédonie, Perpignan, France; 4 Laboratoire d’Excellence “CORAIL”, Paris, France; 5 PSL Université Paris, USR CRIOBE - EPHE-UPVD-CNRS, Perpignan, France; University of the Ryukyus, JAPAN

## Abstract

Understanding the processes that maintain coral assemblages is of crucial importance given increasing rates of coral mortality on reefs globally. Here, we compared relationships among distribution patterns of recruit, juvenile, and adult corals with distinct life history traits to determine the contribution of early life stages to the structure of adult assemblages at Toliara, southwest Madagascar. Results highlighted a marked spatio-temporal variability in the abundance of all life stages within and between major reef habitats. Indications of stock-recruitment relationships (where the adults drive the abundance of early life stages) were found for Acroporidae, whereas Poritidae and its dominant genus *Porites* were likely regulated by recruitment-limitation mechanisms (where early life stages drive the abundance of adults), with significant correlations between the abundance of juveniles and those of adults of the subsequent years. We found stronger links between all life stages for Pocilloporidae, indicative of both recruitment-limitation and stock-recruitment relationships. In contrast, no significant correlations were recorded for the category of ‘other’ families, which is likely the result of mixing taxa with different life history traits. In fact, positive correlations between juveniles and adults were found for *Galaxea*, *Cycloseris*, and *Pavona* genera, which made up the ‘other’ category. The discrepancies of regulation processes among coral taxa highlighted here suggest implementing conservation actions that benefit all life stages. Maintaining the biomass of herbivorous fishes and invertebrates to control algal biomass can benefit coral recruitment and decrease mortality of early life stages and adult colonies. Our results also suggest that sites on the outer slope and on patch reefs, which show higher recruitment rates and abundance of adult colonies, could be considered as recruitment hotspots.

## 1. Introduction

Coral reefs are crucial ecosystems in terms of biodiversity and productivity. They protect coastlands and provide goods and services that contribute to the well-being of ~850 million people from more than 100 countries [1,2]. However, coral reefs are threatened by large-scale disturbances and local stressors, such as thermally induced bleaching events, cyclones, extracting activities, coastal development, algal blooms, and predators and disease outbreaks, that have increased in frequency and severity in the last five decades [3–8]. Scleractinian corals, the primary framework builders of the reef ecosystem and key components of coral reef health and diversity, have been particularly affected with, for example, severe decline of coral cover and abundance, and reduced coral growth, fecundity and recruitment [9–13]. This degradation of coral communities and reef habitats has also caused phase shifts in community structure, with the replacement of corals by algae or other non-reef-building benthic organisms, challenging the ecological function and the goods and services of reef ecosystems [8,14]. As recovery of coral assemblages following disturbances is mainly driven by the settlement of new larval recruits, the widespread mortalities of corals are also challenging the maintenance and resilience of several coral reefs worldwide [15–19].

Corals, like many other marine invertebrates, have a complex bipartite life cycle, which includes a pelagic larval phase followed by a sessile benthic phase for most species. Corals have complex reproductive strategies that differ among coral taxa as well as among regions and habitats within the same taxa [11]. Broadcast spawners, the dominant reproductive mode in corals, release large quantities of gametes into the water column for external fertilization and development, occurring seasonally and generally once a year, while brooders produce fewer offspring, with several spawning events per year for some species [11]. Most corals require several years of benthic life to become sexually mature adults [20–22]. Consequently, the recruitment phase includes the recruit (corals a few weeks/months old, less than 1 cm in diameter, difficult to see with the naked eye) and the juvenile (corals typically aged at least 1 year, 1 < ∅ < 5 cm) stages [23–25]. The presence of recruits largely reflect the variability in larval supply, whereas juveniles tend to represent successive cohorts and reflect the short-term history of settlement combined with early post-settlement mortality [20,22,26]. Recruitment is a critical process in the spatial patterns and dynamics of local coral assemblages, and in recovery following disturbances [10,19,24,27,28]. Several extrinsic physico-chemical factors such as light intensity, sedimentation, substrate characteristics, and water quality may affect coral recruitment patterns [25,29–32]. Hydrodynamic patterns also play a key role in coral recruitment processes, as strong regional currents likely facilitate larval dispersal and connectivity among local and regional populations, and their subsequent recruitment rates [30,31]. In addition, biotic interactions such as predation, allelopathy, and competition with algae also influence the growth and survivorship of small corals [33,34]. Chemical cues from organisms such as crustose coralline algae may enhance settlement of some coral larvae [35–41]. For most coral species, post settlement mortality is generally high until colonies reach an adequate size to withstand competition with other benthic organisms and predation by corallivores [20,42,43].

The contribution of early recruits and post-settlement events on the structure and dynamics of adult populations generally varies among coral taxa with contrasting life history strategies, though spatial variation arises at multiple scales in relation to site-specific environmental conditions, notably the intensity of spatial competition and predation [23,43–45]. A positive correlation between spatial distribution of recruits/juveniles and adults is considered an indication of either recruitment-limitation relationships, where early life stages drive the abundance of adults, or stock-recruitment relationships, where the adults drive the abundance of recruits [44,46–48]. In contrast, dissimilarities between recruits and adults likely suggest that patterns established at settlement may be modified by variable post-settlement mortality through competition and predation, or can reflect the contrasting effects of environmental stressors on these distinct life stages [9,49,50]. Supporting data for stock-recruitment or recruitment-limitation relationships, or alternative models with a predominance of predation-competition have been proposed for various coral reefs [20,27,51–59]. However, most of these studies have either compared recruits and adults, or juveniles and adults; few have analyzed all three stages (but see [20,45]), thus limiting our understanding of mechanisms of population regulation for organisms such as corals with long and complex life cycles [43,60].

With ~2400 km^2^ of coral reefs along 1400 km of coastline, Madagascar is a hotspot of biodiversity in the South Western Indian Ocean (SWIO). These reefs, and particularly those on the southwest coast, provide an important source of food and income for human populations. Coral reefs surrounding the island have been confronted by several large-scale disturbances, most notably the bleaching events of 1998 and 2015–2016, along with local stressors such as sedimentation, overfishing and gleaning activities [61,62]. A decline in coral cover and abundance has been documented in the last 50 years, with coral cover decreasing from ~50% in the 1960s to 5% in the 1980s [61–63]. Though recent advances have been made on characterizing the spatial distribution of coral assemblages at local and regional scales [63–66], including information on juveniles [59,67–70] and recruits [71,72], the regulation of local coral populations through the combined analysis of the three main life stages (recruits, juveniles, and adults) remains unexplored. This type of information is not only crucial to better understand population maintenance and dynamics [15,17,73,74], but also to determine appropriate conservation measures urgently needed for these reefs.

To examine population regulation processes in the region of Toliara, we collected a comprehensive and original data set comparing coral abundance of the three life stages among and within major reef habitats over three consecutive years. In Botosoamananto et al. [72], we examined the spatial and temporal patterns of coral recruitment. Here, we compared relationships among distribution patterns of recruit, juvenile, and adult corals with distinct life history traits to determine the contribution of early life stages to the structure of adult assemblages. Implications of our results for conservation and management of these reefs are also discussed.

## 2. Materials and methods

### 2.1. Study area

This study was conducted in the Toliara region, southwest Madagascar, including the Great Reef of Toliara (GRT), one of the largest reef complexes of the region. The region has two main seasons: the austral summer from October to March is a warm season with occasional rains and tropical cyclones, and the austral winter between April to September corresponds to the cooler and dry season [75]. Sea surface temperature falls to 18°C during the winter season and rises to 30°C during the warm season. Coral bleaching associated with El Niño events occurred in 1998–1999 and 2015–2016, causing substantial damage to several reefs of the region. Cyclones are less frequent in the southwest coast compared to the eastern coast of Madagascar or to other regions in the SWIO, and the last major cyclone, Haruna, passed 120 km north of Toliara in February 2013 [59] with no reported significant impacts on the GRT. Outbreaks of the coral predator *Acanthaster* spp. have not been reported in recent years on the GRT. Dominant winds are from southwest direction. The total annual rainfall is ~ 400 mm. Two main rivers, Fiherenana in the north and Onilahy in the south, contribute the high sedimentation discharge in the region [75].

### 2.2. Sampling strategy

Ten study stations were located between the village of Ifaty in the north and the village of Sarodrano in the south, on the three major reef habitats: four stations on patch reefs (PR1 to PR4), two stations on the inner slope (IS1 and IS2), and four stations on the outer slope (OS1 to OS4). Station codes are abbreviated as follows: the first two letters represent the habitats (PR: patch reefs, IS: inner slope, and OS: outer slope) and the number specifies each station. Stations were located between 7 and 12 m in depth to match those that were established to survey the diversity, abundance, and cover of adult coral assemblages (see Botosoamananto et a. [65] for more information on station locations). Each station represents an area of ~100 m^2^ that includes recruitment tiles and belt-transects for juvenile and adult coral sampling.

Recruits were sampled using 11 × 11 × 1 cm unglazed terracotta tiles attached horizontally to the substratum with a stainless-steel mounting plate [76,77]. At each station, 20 tiles were deployed for a period of four months (October to January) for three consecutive years (2018–2019, 2019–2020, and 2020–2021). At retrieval, tiles were plunged into a bleach solution and rinsed in freshwater to remove all living tissues and sand. In the laboratory, recruits were counted and identified at the family level. At this stage of development, only three families (Acroporidae, Pocilloporidae, Poritidae) could be distinguished, and all other recruits were compiled into a category named ‘other’ recruits (S1 Fig in S1 File) [78]. Recruits that were too damaged to be identified with certainty were not categorized as they represented < 4% of the recruits, but were however added in the overall recruitment counts (all categories pooled). See Botosoamananto et al. [72] for further details on sampling of coral recruits.

Abundance of juvenile (1 < ∅ < 5 cm) and adult (∅ ≥ 5 cm) corals at the genus level was estimated at each station between July and August each year from 2018 through 2020. At each station, three randomly replicated belt-transects of 10 m^2^ (10 × 1 m), laid parallel to depth contours and separated by 1 m [20] were used to sample coral colonies. All juvenile and adult colonies visible without removing sand, debris or other organisms, and with >50% of their living tissue area contained within each belt-transect were recorded without time limitation by the same observer at all stations. See Botosoamananto et al. [65] for further details on sampling of adult corals.

This study was designed and carried out with the agreement and permission of the University of Toliara and the Madagascan Ministry of Higher Education and Research (MESupReS).

### 2.3. Data analysis

Spatial and temporal variation in the abundance of recruit, juvenile, and adult colonies were explored using a negative binomial error structured Generalized Linear Mixed Model (GLMM), with nested fixed factors such as years, habitats within years, and stations within habitats. We used the lme4 package [79] in R [80] for our analyses. We conducted separate analyses for recruits, juveniles, and adults of the five categories (all taxa, Acroporidae, Pocilloporidae, Poritidae, and ‘other’ taxa). Our final model with stations nested within habitats as random factors was selected based on Akaike Information Criterion (AIC).

Since the data did not meet the assumption for parametric tests, Spearman rank correlations were used to analyze the relationships between recruit, juvenile, and adult abundances. Separate analyses were conducted for all taxa, Acroporidae, Pocilloporidae, Poritidae, and ‘other’ taxa. We calculated correlations between abundance of recruits and of the following year’s (year + 1) abundance of juveniles (e.g., recruits 2018–2019 with juveniles 2019) and of adults of year + 2 (e.g., recruits 2018–2019 and adults 2020) to examine potential recruitment-limitation relationships. We also calculated correlations between abundance of adults and abundance of recruits of the following reproductive season (e.g., adults 2019 with recruits 2019–2020) and abundance of juveniles of years + 1 and + 2 (e.g., adults 2018 with juveniles 2019 and juveniles 2020) for potential stock-recruitment relationships. To examine such relationships at a finer taxonomic scale than families, we also calculated the Spearman rank correlations between abundance of juveniles and adults of the eight dominant genera. All analyses were performed in R 4.1.0 [80].

## 3. Results

### 3.1. Variation in the abundance of coral colonies

**Fig 1:**
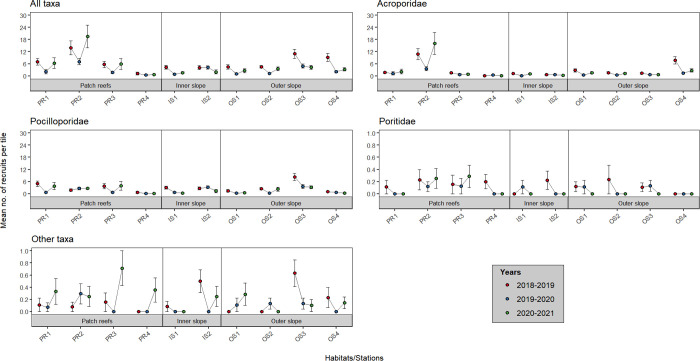
Spatial and temporal variation of the mean abundance of coral recruits at the 10 stations located on the three major habitats, for the five categories (all taxa pooled, Acroporidae, Pocilloporidae, Poritidae, and ‘other’ taxa). Error bars represent standard error.

Recruit assemblages (all stations/years pooled) were dominated by Acroporidae (45.5%) and Pocilloporidae (45.0%), whereas the relative abundance was much lower for Poritidae (1.9%) and ‘other’ recruits (3.6%). A total of 1715 recruits were recorded during the three years of the study, and abundance of recruits was higher in 2018–2019 (6.27 ± 0.59 recruits.tile^-1^, mean ± SE, representing 219.20 recruits.m^-2^), compared to 2019–2020 (2.71 ± 0.33 recruits.tile^-1^, representing 94.75 recruits.m^-2^), and 2020–2021 (4.47 ± 0.77 recruits.tile^-1^, representing 156.30 recruits.m^-2^; S1 and S4 Tables in S1 File). Recruitment rates were variable among habitats and stations, with slightly higher recruit abundance on patch reefs (5.35 ± 0.70 recruits.tile^-1^), compared to the outer slope (4.49 ± 0.42 recruits.tile^-1^) and the inner slope (3.09 ± 0.39 recruits.tile^-1^; Fig 1, S1 Table in S1 File). A higher value of recruit abundance was recorded at station PR2 in 2020–2021, whereas station PR4 was characterized by lower recruitment rates compared to other patch reef stations, with no significant temporal trend. At stations PR1 and PR3, the decrease in recruitment rates between 2018–2019 and 2019–2020 was followed by a return to initial values in 2020–2021. Except station IS2, inner and outer slope stations also showed a decrease in recruitment rates between 2018–2019 and 2019–2020, followed by a slight increase in 2020–2021, except at OS3.

A total of 38 and 45 genera were recorded for juvenile and adult corals, respectively, and a significant variation of generic richness was found among habitats and stations, while no significant temporal variation was recorded (S2 Fig, S2 to S5 Tables in S1 File). Juvenile and adult assemblages were dominated by eight genera that contributed to 63.2% of the total colonies recorded: *Acropora* (16.3%), *Seriatopora* (9.7%), *Porites* (8.4%), *Pocillopora* (6.7%), *Galaxea* (6.3%), *Cycloseris* (5.8%), *Pavona* (5.3%), and *Montipora* (4.7%). During the three years, a total of 5522 juvenile (representing 62.04 ± 5.03 colonies.10 m^-2^) and 12323 adult (138.46 ± 9.29 colonies.10 m^-2^) colonies were recorded.

**Fig 2:**
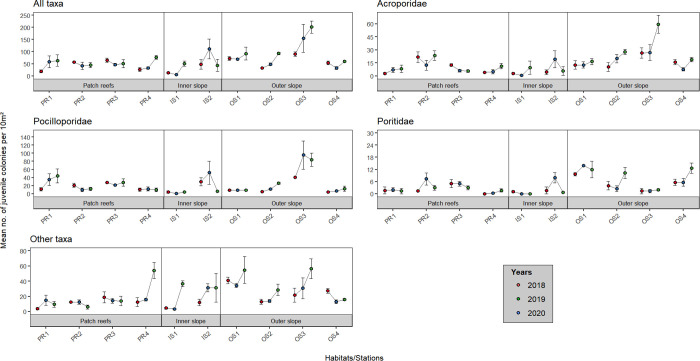
Spatial and temporal variation of the mean abundance of juvenile corals at the 10 stations located on the three major habitats, for the five categories (all taxa pooled, Acroporidae, Pocilloporidae, Poritidae, and ‘other’ taxa). Error bars represent standard error.

**Fig 3:**
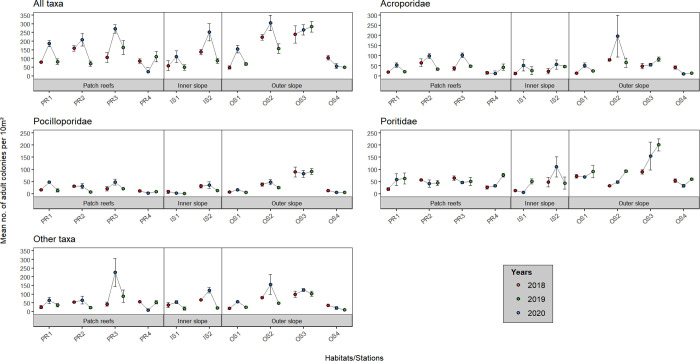
Spatial and temporal variation of the mean abundance of adult corals at the 10 stations located on the three major habitats, for the five categories (all taxa pooled, Acroporidae, Pocilloporidae, Poritidae, and ‘other’ taxa). Error bars represent standard error.

Mean colony abundance of both juvenile and adult assemblages was highly variable among habitats and stations, for all categories of corals (all taxa pooled, Acroporidae, Pocilloporidae, Poritidae, and ‘other’ taxa; Figs 2 and 3, S2, S3, S6 and S7 Tables in S1 File). The highest mean juvenile and adult abundances were recorded at the outer slope (84.45 ± 9.61 colonies.10 m^-2^ and 159.37 ± 16.72 colonies.10 m^-2^, respectively), notably at stations OS3 for juveniles and OS2 and OS3 for adults, while the lowest values were found at the inner slope (45.27 ± 11.10 colonies.10 m^−2^ and 150.44 ± 19.20 colonies.10 m^−2^ for juveniles and adults, respectively), notably at station PR4. For juvenile corals, an increase in abundance was recorded the third year (2020) at the outer slope, whereas for adults, abundance values were highest in 2019 at all stations except PR4.

### 3.2. Relationships between recruit, juvenile, and adult corals

**Fig 4:**
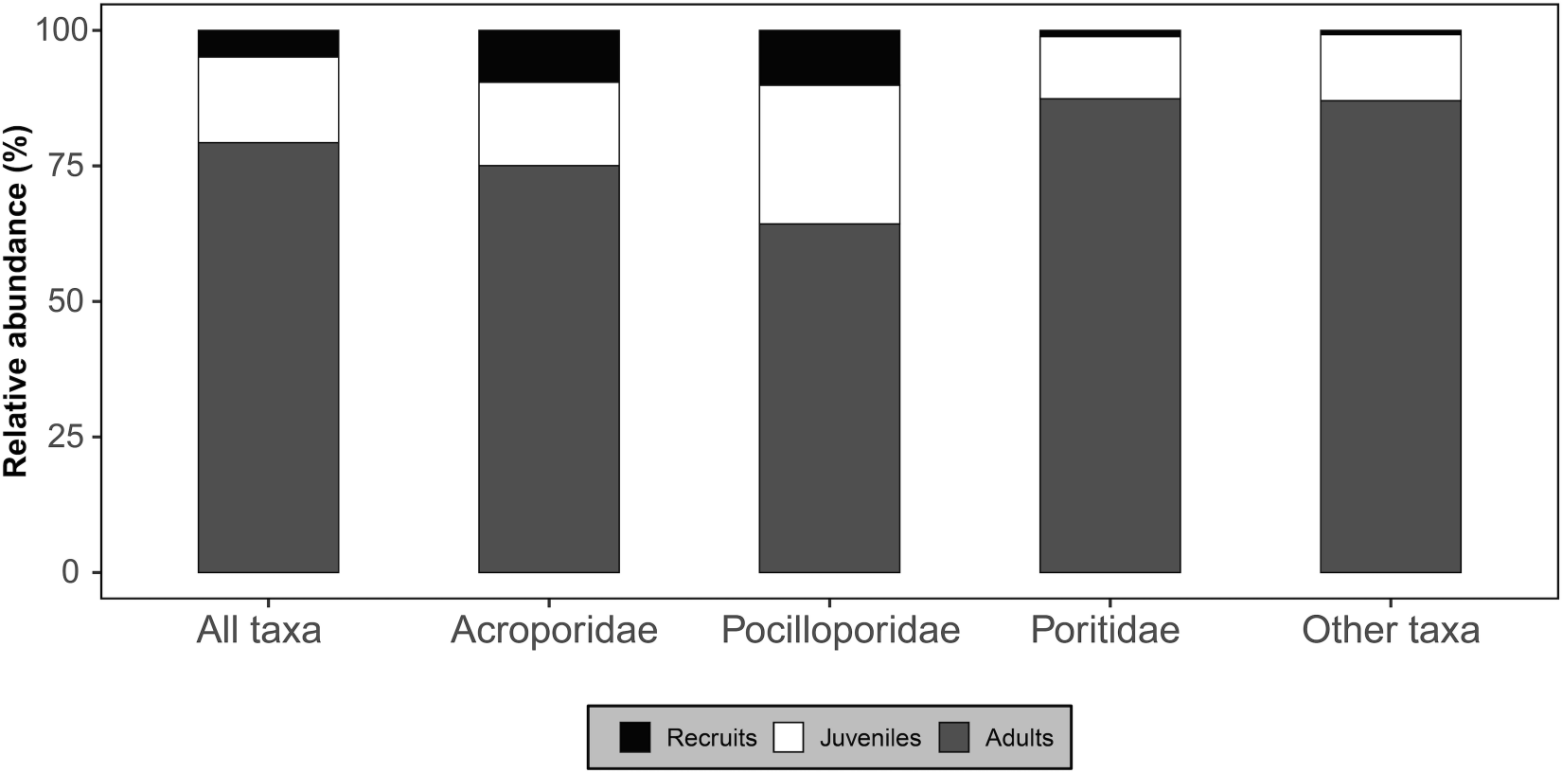
Relative abundance (%) of recruit, juvenile and adult colonies for each of the five categories (all taxa pooled, Acroporidae, Pocilloporidae, Poritidae, and ‘other’ taxa). Mean of the three years and the 10 stations.

The relative abundance of the three life stages varied among families (Fig 4), with the highest proportion of recruits being for Pocilloporidae (10.1% of Pocilloporidae across life stages) and Acroporidae (9.6%), compared to Poritidae (1.2%) and ‘other’ taxa (0.8%) for which recruits represent a very low proportion of total colonies. The proportion of juveniles also differed among coral families, with a higher contribution for Pocilloporidae (25.6%), but with less variability between Acroporidae (15.4%), Poritidae (11.5%), and ‘other’ taxa (12.2%). The proportion of adults in the local assemblages was slightly higher for Poritidae (87.3%) and ‘other’ taxa (87.0%), compared to Acroporidae (75.0%) and Pocilloporidae (64.3%).

When looking at relationships between the three life stages for specific years (Table 1, S3-S7 Figs in S1 File), no significant correlations were recorded between abundance of recruits and juveniles for all taxa pooled, Acroporidae, Poritidae, and ‘other’ taxa, or between recruits and adults for Poritidae and ‘other’ taxa, or between juveniles and adults for ‘other’ taxa. For all taxa pooled, a significant correlation was recorded between abundance of adults in 2018 and recruits in 2018–2019, indicative of a potential stock-recruitment relationship. Such relationships were also signaled for Acroporidae, with positive and significant correlations between adults in 2018 and recruits in 2018–2019 and between adults in 2018 and juveniles in 2020, and for Pocilloporidae, with correlations between adults in 2018 and recruits in 2018–2019, adults in 2020 and recruits in 2020–2021, and adults in 2018 and juveniles in 2019. Recruitment-limitation relationships were suggested by positive and significant correlations between recruits in 2018–2019 and adults in 2020 for Pocilloporidae, and between juveniles in 2018 and adults in 2019 and 2020 for Pocilloporidae and Poritidae (Table 1).

**Table 1:** Correlations between abundance of recruit, juvenile, and adult colonies for the five categories of corals (all taxa, Acroporidae, Pocilloporidae, Poritidae, and ‘other’ taxa).

	Recruits						Juveniles					
	2018−19		2019−20		2020−21		2018		2019		2020	
	ρ	p	ρ	p	ρ	p	ρ	p	ρ	p	ρ	p
**All taxa**												
Juveniles 2019	0.34	ns										
Juveniles 2020	0.15	ns	−0.11	ns								
Adults 2018	**0.82**	^**^							0.35	ns	0.16	ns
Adults 2019			0.52	ns			0.50	ns			−0.16	ns
Adults 2020	0.18	ns			0.06	ns	0.44	ns	0.36	ns		
**Acroporidae**												
Juveniles 2019	0.35	ns										
Juveniles 2020	0.62	ns	0.48	ns								
Adults 2018	**0.69**	^*^							0.58	ns	**0.63**	^*^
Adults 2019			0.21	ns			0.42	ns			0.28	ns
Adults 2020	0.12	ns			−0.40	ns	0.50	ns	0.41	ns		
**Pocilloporidae**												
Juveniles 2019	**0.69**	^*^										
Juveniles 2020	0.40	ns	0.31	ns								
Adults 2018	**0.63**	^*^							**0.78**	^**^	0.47	ns
Adults 2019			0.60	ns			**0.91**	^***^			0.48	ns
Adults 2020	**0.69**	^*^			**0.85**	^***^	**0.62**	^*^	**0.68**	^*^		
**Poritidae**												
Juveniles 2019	0.13	ns										
Juveniles 2020	0.01	ns	0.07	ns								
Adults 2018	−0.05	ns							0.46	ns	0.47	ns
Adults 2019			−0.17	ns			**0.79**	^**^			0.39	ns
Adults 2020	−0.09	ns			0.00	ns	**0.64**	^*^	0.39	ns		
**Other taxa**												
Juveniles 2019	0.16	ns										
Juveniles 2020	−0.01	ns	**−**0.05	ns								
Adults 2018	0.25	ns							0.15	ns	0.33	ns
Adults 2019			0.20	ns			0.22	ns			0.06	ns
Adults 2020	−0.10	ns			0.09	ns	0.23	ns	0.38	ns		

Only meaningful correlations that may represent potential stock-recruitment, recruitment-limitation, and predation-competition relationships are presented (e.g., there is no ecological sense to calculate a correlation between recruits recorded in October 2018 – January 2019 and adults recorded in July-August 2019). Spearman rank correlation coefficients (ρ) and the associated *p*-values (ns: p > 0.05;

*: p < 0.05;

**: p < 0.01;

***: p < 0.001) are given.

Relationships between juvenile and adult abundances of the eight dominant coral genera confirm the weak relationships among juveniles and adults for Acroporidae, with only one significant correlation between adults in 2019 and juveniles in 2020 for *Montipora* (Table 2, S8-S16 Figs in S1 File). For Pocilloporidae, we recorded significant correlations between all combination of juveniles and adults for *Seriatopora*, with no such correlation recorded for *Pocillopora*. For Poritidae, the significant correlation between juveniles in 2018 and adults in 2019 and 2020 is largely driven by *Porites*, the only dominant genera of this family. For ‘other’ taxa, we recorded positive correlations between all combinations of years for juveniles and adults *Galaxea*, and for some years for *Cycloseris* and *Pavona*, although no correlations were recorded when pooling these three genera and the other 30 that composed the category ‘other’ taxa (Table 1).

**Table 2:** Correlation between abundance of juvenile and adult corals of the overall assemblage (all genera pooled) and for the eight dominant genera (*Acropora*, *Montipora*, *Pocillopora*, *Seriatopora*, *Galaxea*, *Porites*, *Cycloseris*, and *Pavona*).

	Juveniles					
	2018		2019		2020	
	ρ	p	ρ	p	ρ	p
**All genera**						
Adults 2018			0.35	ns	0.16	ns
Adults 2019	0.50	ns			−0.16	ns
Adults 2020	0.40	ns	0.36	ns		
**Acroporidae**						
***Acropora***						
Adults 2018			0.55	ns	0.48	ns
Adults 2019	0.07	ns			−0.26	ns
Adults 2020	0.24	ns	0.30	ns		
***Montipora***						
Adults 2018			−0.16	ns	0.26	ns
Adults 2019	0.00	ns			**0.67**	^*^
Adults 2020	0.60	ns	0.21	ns		
**Pocilloporidae**						
***Pocillopora***						
Adults 2018			0.14	ns	0.33	ns
Adults 2019	0.42	ns			0.39	ns
Adults 2020	0.54	ns	0.05	ns		
***Seriatopora***						
Adults 2018			**0.87**	^***^	**0.72**	^*^
Adults 2019	**0.97**	^***^			**0.76**	^*^
Adults 2020	**0.88**	^***^	**0.95**	^***^		
**Poritidae**						
***Porites***						
Adults 2018			0.53	ns	0.10	ns
Adults 2019	**0.75**	^*^			0.31	ns
Adults 2020	**0.74**	^*^	0.54	ns		
**Other taxa**						
***Galaxea***						
Adults 2018			**0.80**	^**^	**0.82**	^**^
Adults 2019	**0.82**	^**^			**0.84**	^**^
Adults 2020	**0.89**	^***^	**0.76**	^**^		
***Cycloseris***						
Adults 2018			**0.70**	^*^	**0.84**	^**^
Adults 2019	0.57	ns			0.61	ns
Adults 2020	**0.77**	^**^	**0.66**	^*^		
***Pavona***						
Adults 2018			0.37	ns	**0.47**	^*^
Adults 2019	0.08	ns			0.29	ns
Adults 2020	0.06	ns	0.47	ns		

Only meaningful correlations that may represent potential stock-recruitment, recruitment-limitation, and predation-competition relationships are presented. Spearman rank correlation coefficients (ρ) and the associated *p*-values (ns: p > 0.05;

*: p < 0.05;

**: p < 0.01;

***: p < 0.001) are given.

## 4. Discussion

### 4.1. Spatio-temporal patterns of coral assemblages

Our results underline the marked spatial variability in the abundance of the three benthic life stages of coral assemblages within and between major reef habitats of the Great Reef of Toliara (GRT) region. Abundances of recruits, juveniles, and adults were higher at several stations of patch reefs, whereas lower values were generally recorded along the inner slope. This strong spatial heterogeneity of corals is consistent with patterns recorded at reefs elsewhere in the world, where marked variation of abundance, together with other coral descriptors such as cover, diversity, and size-structure are documented within and between reef habitats [20,22,25,65,81–84]. Although outside the scope of our survey, this heterogeneity is likely resulting from the marked variability of the environmental conditions and anthropogenic stressors in the GRT [65]. Previous studies have highlighted higher fishing efforts, notably using destructive practices such as mosquito net trawl, beach seine, coral-turning fishing, or fishing by poisoning, as well as higher sedimentation on the inner slope of the GRT [75,85–87]. Overfishing and sedimentation also impact the patch reefs and the outer slope in this area, but to a lesser extent the coral assemblages, likely due to the higher hydrodynamic and greater depths in these habitats compared to the inner slope [65]. Moreover, higher cover of turf and macroalgae at inner slopes likely reduce the abundance of corals through spatial competition [65]. The higher diversity and cover of coral assemblages recorded along the outer slopes and patch reefs are also explained by the more favorable environmental conditions along these sites [65]. For coral recruits, the importance of benthic components on the spatial patterns has also been largely documented [33,36,38,39]. On the GRT, cover of algae and other living taxa such as sponges have been identified as important drivers of the spatial patterns of coral recruitment [72]. The higher cover of algae on the inner slopes likely inhibits larval settlement and increases the post-settlement mortality. Moreover, macroalgae have negative physical effects on early life stages of corals through abrasion, shading, and smothering [88]. In contrast, higher hydrodynamic and water circulation, present along outer slopes and patch reefs, creates more favorable conditions for coral recruitment [20,49]. However, these potential controlling environmental factors of the spatial variability of coral assemblages should be more closely examined, for example through dedicated water quality monitoring surveys in the GRT. Such environmental surveys will not only improve our understanding of the spatial patterns and dynamics of coral assemblages and other reef communities, but will also help identify which threats to prioritize reducing for better conservation and management of these reefs.

Our survey also highlighted a marked interannual variation in the abundance of recruit, juvenile, and adult coral colonies. However, the temporal patterns differed between the three life stages. Recruits were most abundant during the first year of the study (2018–2019), whereas highest abundances for adults were generally recorded in 2019, and in 2020 for juveniles. The interannual changes in coral assemblages are often associated with either large-scale disturbances, such as cyclones, thermally induced bleaching events, or predator outbreaks, or to local stressors, such as sudden deterioration of substrate composition or water quality [10,25,31]. For coral recruitment, a large part of the temporal variability is also linked to variation in fecundity of adult colonies, which is driven by seasonal and interannual changes in climatic and oceanographic conditions [9,30,44]. However, during the study period, no such disturbances or stressors can be associated with the observed temporal changes in coral abundance. Elevated sea surface temperatures (~31°C) were recorded in January and February 2020 [72], but caused neither a significant reduction in recruitment rates the following year (October 2020–January 2021) nor a decrease in juvenile and adult corals the following months (July–August 2020). Rigorous data collection on other potential drivers of the temporal changes in the abundance coral assemblages, such as interannual variation in sedimentation discharges by the two main rivers, should be pursued and examined to determine influence.

### 4.2. Regulation of coral assemblages

Similar to studies of coral reefs in other regions, our results identify discrepancies among families and genera for population regulation processes within the same area, likely due to differences in life history traits and strategies such as reproductive modes, stress tolerance, growth capacities, or competitive abilities [20,23,30,54,55,89]. When examining overall coral assemblages (all taxa pooled), the links between spatial variation of the three life stages (recruits, juveniles, and adults) were weak, with only one significant correlation between abundance of adult corals and recruits in the following reproductive season suggesting a stock-recruitment relationship. This lack of strong and consistent correlation likely results from the mixing of various coral taxa with contrasting life history traits and population dynamics, but may also reflect the contrasting effects of various environmental stressors on these distinct life stages [9,49,50].

For Acroporidae, indications of stock-recruitment relationships were found, with the abundance of adults correlated with those of recruits and juveniles of subsequent years, with no evidence recorded for recruitment-limitation relationships (i.e., when early life stages drive the abundance of adults). The juvenile-adult relationship was restricted to *Montipora*, whereas no significant correlation was recorded for *Acropora*, the other dominant genus of this family. The limited positive correlations between early life stages and adults may be partially explained by the high rate of asexual reproduction through fragmentation in this family, which may mask potential recruitment-limitation relationships, by increasing the number of adult colonies on the reef without sexual reproduction [90,91].

In contrast, we recorded indications of recruitment-limitation relationships for Poritidae corals and its dominant genus *Porites*, although restricted to significant correlations between the abundance of juvenile stages and those of adults of the subsequent years. The lack of positive correlations between recruits and other life stages may be explained by the reproductive strategies of this taxa, as most *Porites* species are brooders that release fewer offspring compared to broadcast spawners corals, such as *Acropora*. Moreover, the extended period of reproduction and spawning of *Porites* in the SWIO region [92,93], which dilutes the larval pool over long periods of the year is likely a large contributor to the low abundance of Poritidae recruits on our recruitment tiles, immersed for four months [72]. As most Poritidae corals are characterized by relatively lower mortality rates and higher resistance to environmental stressors after reaching a certain size [91], our results suggest that most juvenile colonies also grow into the adult stage in reef habitats of the Toliara region.

For Pocilloporidae, we found strong correlations between all three life stages, indicative of both recruitment-limitation and stock-recruitment relationships, that are likely driven by *Seriatopora*. This family is generally characterized by high recruitment rates and relatively low mortality rates, except during occasional acute stressors such as bleaching events [91], which may explain the correlation between the abundance of early life stages and adult colonies. Moreover, the positive correlations between early life stages and adult colonies may also result from self-seeding occurring at the local scale [54,94]. In fact, the most abundant species of Pocilloporidae in the reefs of Toliara include brooders *Pocillopora damicornis*, *Seriatopora hystrix*, and *Stylophora pistillata*, which release larvae with short competency periods that often settle near their parents [95,96].

On the other hand, no significant correlations were recorded between life stages for the category ‘other’, which is again likely the result of mixing various families with different life history traits and variable tolerance to environmental stressors. In fact, the positive correlations between juveniles and adults recorded for *Galaxea*, *Cycloseris*, and *Pavona*, the three dominant genera which make up this ‘other’ families category, clearly indicate that this latter category contains coral taxa with contrasting life history strategies. For those coral taxa that failed to show significant positive correlations between early life stages and adult corals, we may assume that the differential post-settlement events are sufficiently strong to distort the pattern established at settlement [24,97–99]. Asexual reproduction through colony fragmentation at our study sites, likely frequent for branching corals, may also mask the association between recruits and adults [27,100]. The relationship between recruits and adults may also break down for coral taxa with long larval duration in large open systems where advection tends to mix larvae from natal and distant reefs, a likely case for *Acropora* corals that are mostly broadcast spawners with long-dispersal larvae [44,101]. Such lack of correlation between early life stages and adult colonies may also reflect the contrasting habitat preferences and the differential effects of environmental stressors on these distinct life stages [58,81,99,100].

Our results have provided some indication of mechanisms of population regulation, that are not only important for a better understanding of the structure and dynamics of coral assemblages, but also for the implementation of appropriate conservation actions. However, this study is based on correlation analyses, and further sampling and field experiments should be conducted to rigorously examine the ultimate causes of the relationships between spatial variation in the abundance of the three life stages of corals. For example, assessment of stock-recruitment relationships should be complemented by monitoring fecundity, density-dependent interactions, and early post-settlement mortality events within local coral populations. Data on reproduction modes and connectivity patterns should also be collected to better examine mechanisms of population regulation, data which are also necessary to estimate recovery capacities and improve conservation actions [102–106]. Moreover, as these relationships may fluctuate with time, the present study should be complemented by a long-term interannual study on the demography of coral assemblages [107].

### 4.3. Implications for conservation strategies

The discrepancies of regulation processes among coral families and genera highlighted here call for conservation actions that include all benthic life stages, rather than specific actions targeting one phase of the coral life cycle. Conservation strategies incorporating life-history processes are likely to be more successful than those based on promoting the abundance of adult corals alone [24]. Thus, the outcomes of this survey suggest implementing conservation actions to increase the settlement rates of coral larvae, reduce early post-settlement mortality, and reduce local threats that affect the health of coral colonies, notably the growth and fecundity of adult colonies. Although some of these actions may be more selective and effective for one life stage, most are in fact beneficial to all coral life stages, as well as other reef communities.

In the context of coral reefs of Toliara, fishing activities are particularly intense, and their management should be a priority [87]. Conservation actions to maintain the biomass and diversity of herbivorous fishes and invertebrates at a sufficient level to control algal biomass is one of the most effective means to promote coral recruitment by offering more adequate substrate for coral larvae to settle [73,108,109]. Reducing algal biomass will also decrease, for both early life stages and adult colonies, mortality caused by spatial competition, abrasion, and allelopathy with fleshy algae [33,110,111]. Destructive fishing methods should also be urgently addressed, as this is the main cause of habitat degradation in the Toliara region [69,86,112]. Practices such as gleaning, mosquito net trawl, and fishing by poisoning, that have direct and indirect negative effects on all coral life stages, should be banned, with alternative income-generating activities, such as aquaculture, proposed to local fishermen [87,113,114].

Local environmental conditions can also be improved by conservation measures regarding land use [115,116], with specific actions to reduce nutrients and sediment loads from the two main rivers, the Fiherenana and Onilahy [86,112]. These actions will benefit overall coral assemblages, as both early life stages and adult colonies are sensitive to increasing levels of sedimentation and nutrients [34,117], and other reef communities.

Given limited human and financial resources, managers are generally constrained to select the most effective areas to protect and the critical periods to minimize stressors. Our results suggest that sites on the outer slope and on patch reefs, which show higher recruitment rates and abundance and cover of adult colonies, could be considered as recruitment hotspots [65,72] to prioritize for protection. Our results also suggest that austral summer, when most corals finalize their gametogenesis and spawn, and when larvae settle and start their benthic life [72], is a critical period when all conservation measures mentioned prior should be particularly reinforced.

However, as coastal human population is largely dependent on reef resources in Madagascar, these conservation measures should be designed and implemented with the strong involvement of end-users through, for example, Locally Marine Managed Areas, which have proven their effectiveness in the Malagasy context, and with the development of alternative sources of income, such as sea cucumber and seaweed farming [113,114,118–120]. Our results, by examining regulation processes of coral assemblages in the region of Toliara, may serve as an important baseline against which future disturbances and recovery trajectories can be measured, and for improving conservation strategies of these highly vulnerable coral reefs.

## Supporting information

S1 File Supplemental Figures and Tables. All supporting figures and tables throughout the text are located in this file.
(PDF)
