# Supplementary material for: Regulation of coral assemblages: Spatial and temporal variation in the abundance of recruits, juveniles, and adults
Source: PLoS One. 2025 Aug 21;20(8):e0329546. doi: 10.1371/journal.pone.0329546 (PMC12370058; doi:10.1371/journal.pone.0329546)
Supplement: S1 File — All supporting figures and tables throughout the text are located in this file. (PDF) [file pone.0329546.s001.pdf]

Regulation of coral assemblages: spatial and temporal variation in the abundance of recruits, juveniles, and adults

Supporting Information

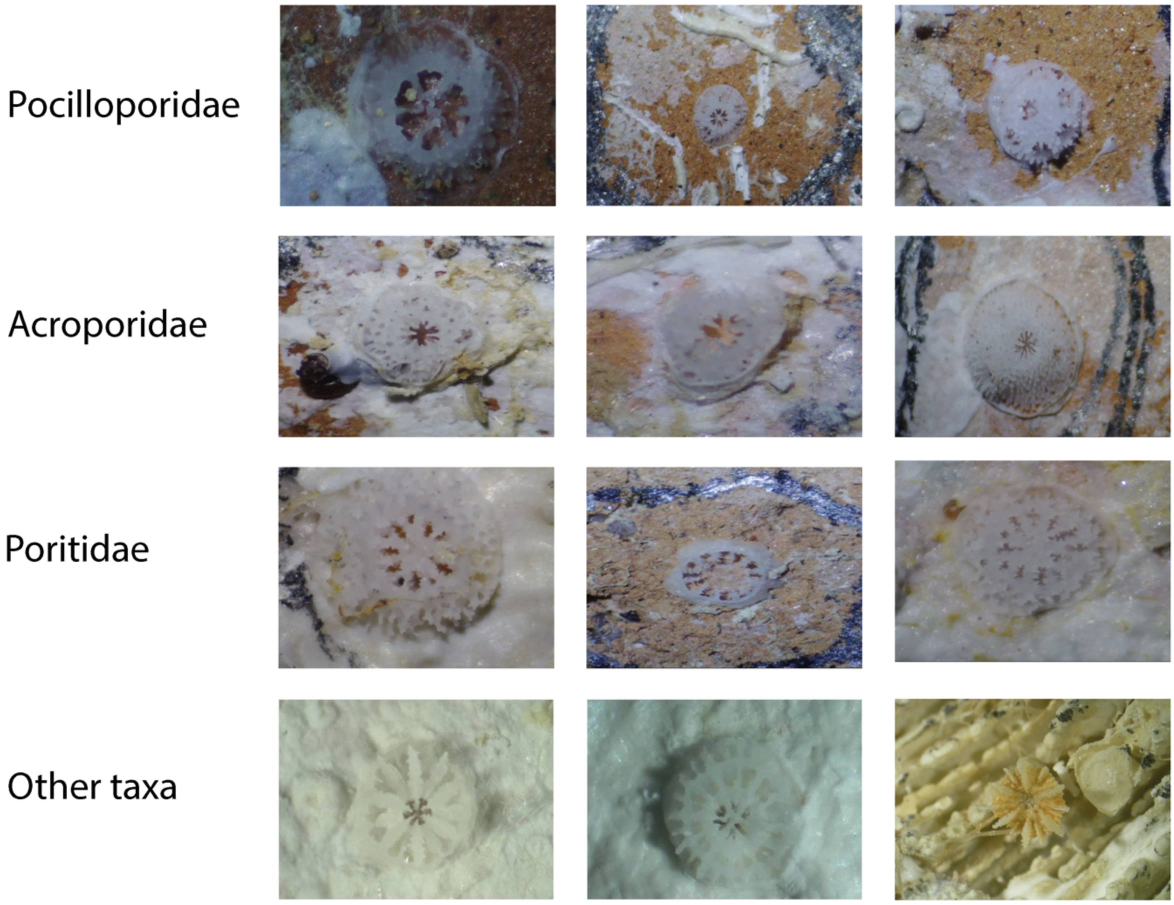

S1 Fig. Photographs of the four categories of coral recruits identified in the present study: Pocilloporidae, Acroporidae, Poritidae, and other taxa.

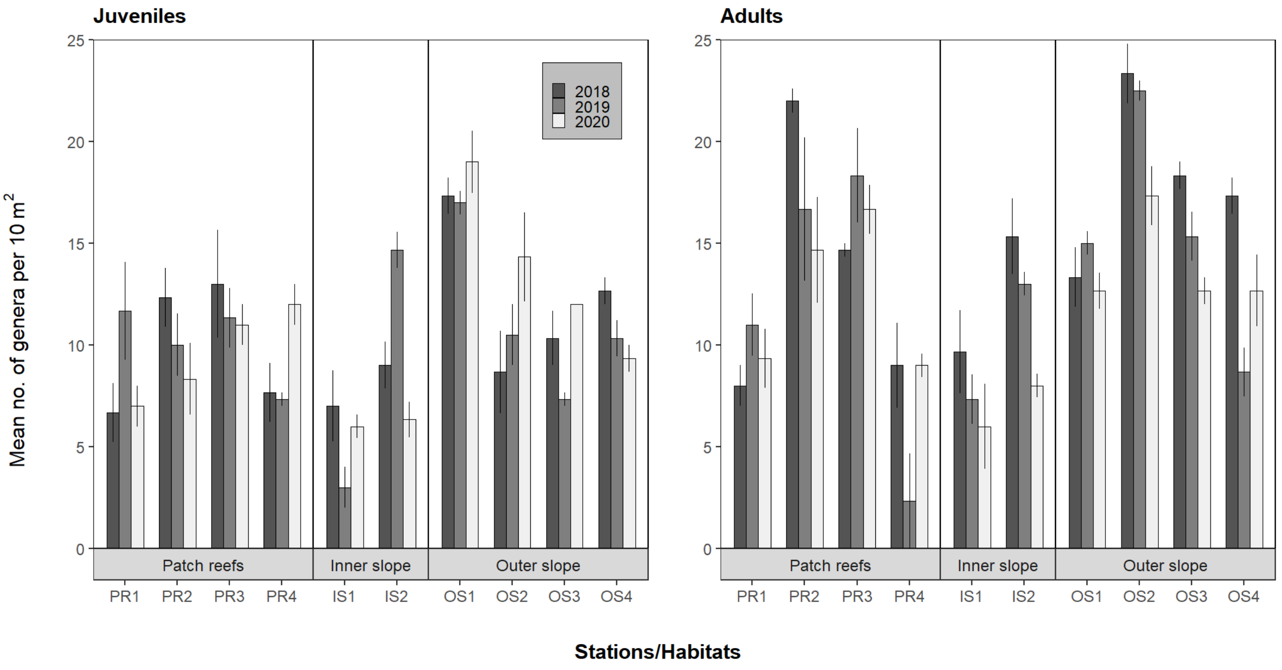

S2 Fig. Spatial and temporal variation of the mean number of genera (juveniles and adults) at the 10 stations located on the three major habitats (PR: patch reefs, IS: inner slope, and OS: outer slope). Error bars represent standard error.

S1 Table. Abundance (mean number of recruits per tile) of coral recruits recorded at the 10 stations during the three years of the study. Standard errors (SE) in brackets.

RECRUITS															
	All taxa			Acroporidae			Pocilloporidae			Poritidae			Other taxa		
Stations	2018	2019	2020	2018	2019	2020	2018	2019	2020	2018	2019	2020	2018	2019	2020
PR1	7.00 (5.02)	2.14 (4.05)	6.33 (6.56)	1.67 (1.22)	1.29 (3.20)	2.17 (2.86)	5.00 (4.12)	0.64 (0.93)	3.83 (4.36)	0.11 (0.33)	0.00 (0.00)	0.00 (0.00)	0.11 (0.33)	0.07 (0.27)	0.33 (0.52)
PR2	13.85 (12.46)	7.06 (6.57)	19.50 (16.07)	10.85 (9.89)	3.65 (4.44)	16.13 (15.32)	1.85 (2.03)	2.65 (2.57)	2.63 (1.41)	0.23 (0.60)	0.12 (0.33)	0.25 (0.46)	0.08 (0.28)	0.29 (0.69)	0.25 (0.46)
PR3	5.69 (5.56)	1.75 (1.49)	5.86 (7.17)	1.54 (1.71)	0.75 (1.04)	0.86 (1.21)	3.69 (4.53)	0.75 (0.71)	4.00 (5.77)	0.15 (0.55)	0.13 (0.35)	0.29 (0.49)	0.15 (0.55)	0.00 (0.00)	0.71 (0.76)
PR4	1.20 (3.11)	0.50 (0.80)	0.64 (0.84)	0.15 (0.49)	0.42 (0.67)	0.07 (0.27)	0.75 (2.51)	0.08 (0.29)	0.14 (0.36)	0.20 (0.52)	0.00 (0.00)	0.00 (0.00)	0.00 (0.00)	0.00 (0.00)	0.36 (0.74)
IS1	4.33 (3.23)	0.89 (0.93)	1.60 (1.51)	1.17 (1.40)	0.11 (0.33)	1.10 (1.10)	3.08 (2.61)	0.67 (0.87)	0.40 (0.52)	0.00 (0.00)	0.11 (0.33)	0.00 (0.00)	0.08 (0.29)	0.00 (0.00)	0.00 (0.00)
IS2	4.17 (4.19)	4.25 (2.38)	2.00 (2.62)	0.61 (1.29)	0.63 (0.74)	0.38 (1.06)	2.67 (3.12)	3.25 (1.75)	1.38 (2.39)	0.22 (0.65)	0.00 (0.00)	0.00 (0.00)	0.50 (0.79)	0.00 (0.00)	0.25 (0.46)
OS1	4.41 (4.44)	1.11 (1.71)	2.67 (2.51)	2.76 (3.35)	0.56 (0.92)	1.57 (1.27)	1.47 (2.48)	0.33 (0.49)	0.43 (0.53)	0.12 (0.33)	0.11 (0.47)	0.00 (0.00)	0.00 (0.00)	0.11 (0.47)	0.29 (0.49)
OS2	4.47 (2.62)	1.27 (1.53)	3.57 (2.44)	1.65 (1.93)	0.53 (0.74)	1.29 (1.11)	2.53 (2.18)	0.40 (0.63)	2.29 (2.63)	0.24 (0.97)	0.00 (0.00)	0.00 (0.00)	0.00 (0.00)	0.13 (0.35)	0.00 (0.00)
OS3	10.95 (10.02)	4.87 (3.96)	4.20 (3.29)	1.32 (1.77)	0.67 (0.90)	0.06 (1.07)	8.37 (7.90)	3.67 (3.66)	3.20 (2.15)	0.11 (0.32)	0.13 (0.35)	0.00 (0.00)	0.63 (0.96)	0.13 (0.35)	0.10 (0.32)
OS4	9.23 (7.32)	2.18 (2.27)	3.29 (2.87)	7.69 (6.41)	1.47 (1.97)	2.71 (2.87)	1.00 (0.91)	0.71 (1.16)	0.29 (0.47)	0.00 (0.00)	0.00 (0.00)	0.00 (0.00)	0.23 (0.60)	0.00 (0.00)	0.14 (0.36)

S2 Table. Abundance (mean number of colonies per 10 m^2) of juvenile corals recorded at the 10 stations during the three years of the study. Standard errors (SE) in brackets.

JUVENILES															
	All taxa			Acroporidae			Pocilloporidae			Poritidae			Other taxa		
Stations	2018	2019	2020	2018	2019	2020	2018	2019	2020	2018	2019	2020	2018	2019	2020
PR1	19.67 (10.21)	59.00 (41.94)	63.33 (39.80)	3.00 (1.73)	7.00 (5.29)	8.33 (7.51)	11.33 (7.37)	35.00 (25.24)	44.00 (29.61)	1.67 (2.89)	2.00 (1.73)	1.33 (2.31)	3.67 (2.52)	15.00 (11.79)	9.67 (6.66)
PR2	56.67 (4.73)	42.00 (26.91)	45.00 (19.05)	21.67 (10.69)	12.33 (10.12)	23.67 (9.45)	21.00 (9.17)	9.67 (7.64)	12.33 (7.23)	1.33 (0.58)	7.33 (5.13)	3.00 (2.00)	12.67 (1.53)	12.67 (5.13)	6.00 (4.36)
PR3	64.00 (16.09)	47.00 (7.94)	50.67 (29.02)	12.67 (2.52)	6.00 (2.65)	5.67 (2.52)	27.67 (2.31)	21.67 (7.64)	28.00 (16.09)	5.00 (3.46)	5.00 (2.00)	3.00 (1.73)	18.67 (12.58)	14.33 (5.51)	14.00 (10.58)
PR4	26.67 (12.06)	33.00 (8.54)	76.67 (12.10)	4.00 (1.00)	5.00 (4.36)	11.33 (5.51)	10.33 (6.51)	12.00 (8.19)	9.67 (7.77)	0.00 (0.00)	0.33 (0.58)	1.67 (1.53)	12.33 (10.12)	15.67 (3.21)	54.00 (18.52)
IS1	13.33 (5.51)	5.00 (1.73)	51.00 (19.08)	3.00 (1.73)	0.67 (0.58)	9.67 (13.28)	4.67 (4.73)	1.00 (1.00)	4.67 (1.15)	1.00 (1.00)	0.00 (0.00)	0.00 (0.00)	4.67 (2.08)	3.33 (1.53)	36.67 (6.35)
IS2	47.67 (33.29)	110.67 (70.78)	44.00 (43.86)	4.33 (4.93)	19.33 (17.10)	5.67 (8.96)	29.67 (17.95)	52.00 (49.52)	6.33 (3.21)	1.67 (2.89)	8.00 (4.36)	0.67 (0.58)	12.00 (7.81)	31.33 (9.07)	31.33 (32.52)
OS1	72.00 (14.11)	69.67 (6.51)	92.33 (44.77)	12.67 (8.50)	12.67 (6.43)	16.67 (6.35)	8.67 (0.58)	8.67 (2.89)	9.00 (1.00)	9.67 (1.53)	14.00 (0.00)	12.00 (7.00)	41.00 (7.81)	34.33 (4.51)	54.67 (30.66
OS2	33.00 (3.61)	48.50 (7.78)	92.67 (9.45)	10.33 (8.74)	20.00 (7.07)	27.67 (6.35)	5.67 (2.31)	12.00 (1.41)	26.00 (5.29)	4.00 (3.61)	2.50 (2.12)	10.33 (4.73)	13.00 (6.00)	14.00 (2.83)	28.67 (12.86)
OS3	90.00 (15.62)	154.67 (99.28)	201.67 (43.25)	26.33 (10.21)	27.00 (15.72)	59.67 (17.79)	40.67 (3.21)	95.67 (60.14)	83.67 (28.29)	1.33 (2.31)	1.33 (1.53)	2.00 (1.00)	21.67 (16.17)	30.67 (23.80)	56.33 (22.68)
OS4	53.67 (12.74)	33.00 (10.15)	60.33 (8.08)	16.00 (5.29)	7.67 (3.06)	19.00 (4.36)	4.67 (1.53)	6.67 (1.15)	12.67 (10.69)	5.67 (2.52)	5.67 (3.21)	12.67 (4.51)	27.33 (5.69)	13.00 (4.36)	16.00 (2.65)

S3 Table. Abundance (mean number of colonies per 10 m^2) of adult corals recorded at the 10 stations during the three years of the study. Standard errors (SE) in brackets.

ADULTS															
	All taxa			Acroporidae			Pocilloporidae			Poritidae			Other taxa		
Stations	2018	2019	2020	2018	2019	2020	2018	2019	2020	2018	2019	2020	2018	2019	2020
PR1	80.00 (4.36)	186.67 (30.29)	82.67 (27.01)	18.67 (4.51)	54.00 (18.33)	21.33 (5.13)	16.00 (2.00)	48.67 (4.16)	14.00 (11.53)	19.33 (10.97)	19.33 (11.02)	10.33 (5.13)	26.00 (17.09)	64.67 (30.55)	37.00 (17.58)
PR2	159.33 (28.43)	208.67 (61.33)	70.67 (25.81)	65.33 (33.26)	98.67 (20.13)	33.33 (7.37)	30.67 (6.66)	32.00 (18.33)	7.33 (6.03)	9.33 (4.51)	12.00 (7.21)	7.00 (2.00)	54.00 (4.36)	66.00 (38.00)	23.00 (11.53)
PR3	106.67 (48.79)	271.33 (42.25)	164.33 (69.00)	36.67 (18.15)	102.67 (18.15)	48.67 (9.07)	21.00 (14.73)	48.00 (22.00)	20.33 (1.15)	6.33 (3.21)	20.00 (16.37)	6.33 (1.53)	42.67 (21.13)	226.00 (141.07)	89.00 (62.51)
PR4	85.67 (21.22)	24.00 (41.57)	111.00 (50.27)	16.33 (12.58)	12.00 (20.78)	43.67 (27.30)	11.67 (8.96)	3.33 (5.77)	8.00 (2.65)	0.00 (0.00)	0.00 (0.00)	6.00 (8.72)	57.67 (5.03)	8.67 (15.01)	53.33 (15.70)
IS1	59.33 (48.64)	111.33 (57.49)	49.00 (26.96)	12.67 (12.01)	52.67 (48.01)	26.67 (34.78)	8.00 (12.12)	2.00 (3.46)	1.67 (2.89)	1.00 (1.00)	2.00 (3.46)	3.67 (4.73)	37.67 (24.58)	54.67 (13.61)	17.00 (16.64)
IS2	138.00 (28.35)	252.67 (86.08)	87.00 (25.24)	23.33 (23.12)	56.67 (39.72)	46.33 (10.02)	31.00 (12.49)	36.00 (24.25)	14.00 (4.00)	15.67 (7.02)	36.67 (13.32)	6.00 (4.58)	68.00 (7.00)	123.33 (27.01)	20.67 (10.41)
OS1	48.67 (16.17)	154.67 (38.28)	68.67 (14.57)	13.33 (4.93)	52.67 (19.63)	24.33 (4.62)	7.00 (3.00)	16.00 (2.00)	6.00 (1.00)	10.33 (2.08)	28.67 (13.61)	12.33 (7.37)	18.00 (10.00)	57.33 (8.33)	26.00 (10.15)
OS2	223.00 (27.73)	306.00 (62.23)	158.00 (47.57)	79.67 (10.26)	197.00 (145.66)	65.67 (38.73)	38.33 (12.66)	48.00 (14.14)	25.00 (8.19)	24.00 (14.11)	49.00 (26.87)	18.33 (3.06)	81.00 (10.54)	157.00 (80.61)	49.00 (7.00)
OS3	239.33 (86.49)	265.33 (51.08)	283.67 (53.53)	49.00 (22.34)	56.00 (12.49)	83.33 (18.45)	89.67 (35.23)	82.00 (24.58)	91.67 (20.60)	0.67 (1.15)	2.67 (3.06)	5.00 (5.00)	100.00 (31.19)	124.67 (15.53)	103.67 (24.79)
OS4	106.00 (23.30)	57.33 (23.18)	50.67 (3.79)	42.33 (14.15)	10.67 (3.06)	14.67 (2.08)	12.67 (6.51)	6.00 (2.00)	6.33 (2.52)	14.33 (2.52)	20.00 (8.00)	19.00 (4.36)	36.67 (6.81)	20.67 (15.01)	10.67 (6.66)

S4 Table. Summary of negative binomial error structured generalized linear mixed effects model, showing the spatial and temporal variability of coral recruitment rates for all taxa, Acroporidae, Pocilloporidae, Poritidae and 'other' recruits. LM: log-mean, CI: Confidence interval.

		**All taxa**		**Acroporidae**		**Pocilloporidae**		**Poritidae**		**'Other' recruits**	
Source of variation	*Predictors*	*LM*	*CI*	*LM*	*CI*	*LM*	*CI*	*LM*	*CI*	*LM*	*CI*
	(Intercept)	**1.22** ***	0.78 – 1.65	0.11	-0.46 – 0.68	**0.73** **	0.22 – 1.24	**-2.07** ***	-2.83 – -1.32	-2.07	-4.20 – 0.07
Years	2019	**-0.79** *	-1.44 – -0.14	**-1.00** ***	-1.47 – -0.53	**-0.77** *	-1.40 – -0.14	-0.89	-1.83 – 0.06	-15.1	-3476.52 – 3446.38
	2020	**-0.66** *	-1.27 – -0.05	**-0.32**	-0.77 – 0.13	**-0.85** *	-1.62 – -0.08	**-1.21** *	-2.42 – -0.01	-0.79	-2.60 – 1.02
Habitats	Outer slope	**0.79** **	0.23 – 1.35	**1.58** ***	0.87 – 2.28	-0.6	-1.32 – 0.12	-0.01	-1.26 – 1.24	0.38	-1.95 – 2.72
	Patch reefs	**-1.14** ***	-1.77 – -0.51	**-1.42** **	-2.34 – -0.51	**-1.40** ***	-2.18 – -0.62	0.57	-0.65 – 1.78	-0.87	-3.16 – 1.42
Stations	IS2	0.43	-0.10 – 0.96	-0.41	-1.18 – 0.37	0.57	-0.04 – 1.18			2.14	-0.03 – 4.31
	OS1	-0.41	-1.18 – 0.36	-0.15	-1.76 – 1.46	-0.16	-1.03 – 0.72			-0.17	-1.58 – 1.24
	OS2	-0.28	-1.05 – 0.48	-0.47	-2.09 – 1.15	0.55	-0.29 – 1.40			-0.82	-2.54 – 0.90
	OS3	0.61	-0.13 – 1.36	-0.68	-2.29 – 0.94	**1.79** ***	0.98 – 2.60			1	-0.13 – 2.14
	PR1	**1.57** **	0.54 – 2.59	**1.65** *	0.08 – 3.22	**1.56** *	0.15 – 2.98			0.47	-1.03 – 1.96
	PR2	**2.58** ***	1.58 – 3.57	**3.22** ***	1.68 – 4.76	**1.67** *	0.25 – 3.09			0.98	-0.30 – 2.26
	PR3	**1.44** **	0.42 – 2.46	1.1	-0.50 – 2.70	**1.59** *	0.19 – 2.99			0.97	-0.38 – 2.32
	OS4	**0.72** **	0.23 – 1.21	**1.53** ***	0.89 – 2.16	-0.63	-1.29 – 0.02				
	PR4	**-1.04** ***	-1.60 – -0.48	**-1.40** **	-2.29 – -0.52	**-1.35** ***	-2.07 – -0.62				
Habitats:Years	Outer slope:2019	-0.6	-1.32 – 0.13	-0.46	-1.64 – 0.71	-0.55	-1.40 – 0.30			16.45	-3694.03 – 3726.94
	Patch reefs:2019	-0.39	-1.15 – 0.37	0.23	-0.97 – 1.43	-0.52	-1.40 – 0.37			17.64	-3692.84 – 3728.13
	Outer slope:2020	0.1	-0.66 – 0.86	-0.57	-1.60 – 0.46	0.42	-0.55 – 1.38			0.5	-1.56 – 2.56
	Patch reefs:2020	**0.79** *	0.01 – 1.56	0.22	-0.85 – 1.29	**0.99** *	0.02 – 1.96			**2.71** *	0.61 – 4.82

* $p<0.05$ ** $p<0.01$ *** $p<0.001$

S5 Table. Summary of negative binomial error structured generalized linear mixed effects model, showing the spatial and temporal variability of generic richness (GR) and abundance of juvenile and adult corals (all taxa pooled). LM: log-mean, CI: Confidence interval.

Source of variation	*Predictors*	**Juvenile GR** *LM*	*CI*	**Adult GR** *LM*	*CI*	**Juvenile abundance** *LM*	*CI*	**Adult abundance** *LM*	*CI*
	(Intercept)	**2.20** ***	1.94 – 2.47	**1.97** ***	1.69 – 2.25	**3.59** ***	3.18 – 3.99	**4.14** ***	3.78 – 4.51
Years	2019	0.02	-0.24 – 0.27	-0.1	-0.33 – 0.12	0.11	-0.29 – 0.52	**0.96** ***	0.59 – 1.32
	2020	-0.03	-0.29 – 0.22	-0.08	-0.30 – 0.15	0.39	-0.01 – 0.80	0.2	-0.16 – 0.57
Habitats	Inner slope	**-0.49** *	-0.93 – -0.04	0.3	-0.11 – 0.70	**-1.01** **	-1.65 – -0.37	**0.73** *	0.17 – 1.29
	Outer slope	0.16	-0.19 – 0.52	**0.72** ***	0.37 – 1.07	0.09	-0.48 – 0.67	**1.21** ***	0.69 – 1.72
Stations	IS2	**0.63** ***	0.28 – 0.98	**0.46** **	0.16 – 0.76	**1.30** ***	0.82 – 1.77	0.13	-0.29 – 0.56
	OS1	**0.50** ***	0.25 – 0.75	0.06	-0.20 – 0.31	**0.47** *	0.00 – 0.93	-0.17	-0.59 – 0.25
	OS2	0.03	-0.26 – 0.32	**0.48** ***	0.24 – 0.72	0.1	-0.38 – 0.58	**0.65** **	0.22 – 1.08
	OS3	-0.09	-0.37 – 0.20	0.18	-0.07 – 0.43	**1.08** ***	0.62 – 1.54	**0.49** *	0.07 – 0.90
	PR1	-0.06	-0.38 – 0.25	**0.33** *	0.00 – 0.66	0.06	-0.40 – 0.53	**0.75** ***	0.33 – 1.18
	PR2	0.13	-0.17 – 0.43	**0.96** ***	0.67 – 1.26	0.14	-0.33 – 0.60	**1.42** ***	1.00 – 1.84
	PR3	0.27	-0.02 – 0.56	**0.89** ***	0.60 – 1.19	0.25	-0.21 – 0.72	**1.39** ***	0.97 – 1.82
Habitats:Years	Inner slope: 2019	0.08	-0.38 – 0.55	-0.1	-0.51 – 0.30	0.23	-0.48 – 0.95	-0.54	-1.17 – 0.09
	Inner slope: 2020	-0.23	-0.73 – 0.27	**-0.50** *	-0.94 – -0.06	0.45	-0.26 – 1.16	-0.58	-1.22 – 0.05
	Outer slope: 2019	-0.1	-0.45 – 0.25	-0.07	-0.37 – 0.23	-0.02	-0.60 – 0.56	0.08	-0.44 – 0.60
	Outer slope: 2020	0.14	-0.20 – 0.48	-0.19	-0.49 – 0.11	0.14	-0.43 – 0.71	**-0.59** *	-1.11 – -0.08

* $p<0.05$ ** $p<0.01$ *** $p<0.001$

S6 Table. Summary of negative binomial error structured generalized linear mixed effects model, showing the spatial and temporal variability of juvenile coral abundance for Acroporidae, Pocilloporidae, Poritidae and 'other' taxa. LM: log-mean, CI: Confidence interval.

		**Acroporidae**		**Pocilloporidae**		**Poritidae**		**Other taxa**	
Source of variation	*Predictors*	*LM*	*CI*	*LM*	*CI*	*LM*	*CI*	*LM*	*CI*
	(Intercept)	**1.85** ***	1.30 – 2.39	**2.33** ***	1.85 – 2.80	-0.69	-1.64 – 0.25	**3.02** ***	2.55 – 3.49
Years	2019	-0.19	-0.72 – 0.35	0	-0.46 – 0.46	0.6	-0.02 – 1.23	0.27	-0.21 – 0.76
	2020	0.25	-0.27 – 0.78	0.14	-0.32 – 0.60	0.19	-0.47 – 0.85	0.38	-0.10 – 0.87
Habitats	Inner slope	**-0.97** *	-1.88 – -0.06	**-0.82** *	-1.61 – -0.04	-0.58	-2.23 – 1.06	**-1.45** ***	-2.22 – -0.68
	Outer slope	0.6	-0.14 – 1.35	-0.77 *	-1.47 – -0.06	**2.47** ***	1.42 – 3.52	-0.14	-0.80 – 0.52
Stations	IS2	**0.78** *	0.13 – 1.43	**1.91** ***	1.30 – 2.52	**2.26** ***	1.00 – 3.51	**0.90** **	0.34 – 1.45
	OS1	0.01	-0.57 – 0.59	0.19	-0.40 – 0.77	0.46	-0.02 – 0.94	**0.80** **	0.28 – 1.33
	OS2	0.26	-0.33 – 0.85	0.56	-0.02 – 1.15	-0.32	-0.87 – 0.22	-0.07	-0.63 – 0.48
	OS3	**0.94** **	0.37 – 1.50	**2.24** ***	1.69 – 2.79	**-1.61** ***	-2.30 – -0.92	**0.60** *	0.07 – 1.12
	PR1	-0.07	-0.71 – 0.57	**1.02** ***	0.48 – 1.55	0.92	-0.10 – 1.93	**-1.03** ***	-1.59 – -0.48
	PR2	**1.06** ***	0.46 – 1.66	0.3	-0.25 – 0.85	**1.72** ***	0.77 – 2.66	**-0.86** **	-1.41 – -0.31
	PR3	0.24	-0.39 – 0.86	**0.88** **	0.34 – 1.42	**1.89** ***	0.95 – 2.82	-0.46	-1.00 – 0.07
Habitats:Years	Inner slope: 2019	0.96	-0.02 – 1.95	0.12	-0.71 – 0.95	0.32	-0.84 – 1.47	0.3	-0.56 – 1.16
	Inner slope: 2020	0.62	-0.35 – 1.59	**-0.89** *	-1.76 – -0.02	-1.68	-3.46 – 0.09	**1.24** **	0.40 – 2.08
	Outer slope: 2019	0.15	-0.59 – 0.89	0.52	-0.16 – 1.20	-0.49	-1.29 – 0.31	-0.43	-1.11 – 0.24
	Outer slope: 2020	0.3	-0.42 – 1.01	**0.69** *	0.03 – 1.36	0.44	-0.37 – 1.25	-0.02	-0.68 – 0.64

* $p<0.05$ ** $p<0.01$ *** $p<0.001$

S7 Table. Summary of negative binomial error structured generalized linear mixed effects model, showing the spatial and temporal variability of adult coral abundance for Acroporidae, Pocilloporidae, Poritidae and 'other' taxa. LM: log-mean, CI: Confidence interval.

		Acroporidae		Pocilloporidae		Poritidae		Other taxa	
Source of variation	*Predictors*	*LM*	*CI*	*LM*	*CI*	*LM*	*CI*	*LM*	*CI*
	(Intercept)	**2.59** ***	2.01 – 3.17	**2.48** ***	2.00 – 2.96	-0.19	-1.03 – 0.65	**3.57** ***	3.22 – 3.93
Years	2019	**1.21** ***	0.64 – 1.77	**1.09** ***	0.63 – 1.55	**1.18** ***	0.52 – 1.85	**0.84** ***	0.49 – 1.19
	2020	0.17	-0.40 – 0.74	0.34	-0.12 – 0.81	**0.81** *	0.14 – 1.48	0.16	-0.19 – 0.52
Habitats	Inner slope	0.77	-0.10 – 1.65	0	-0.74 – 0.74	**2.46** ***	1.33 – 3.59	**0.85** **	0.31 – 1.39
	Outer slope	**0.84** *	0.03 – 1.65	0.19	-0.48 – 0.87	**3.55** ***	2.51 – 4.60	**1.27** ***	0.77 – 1.77
Stations	IS2	-0.31	-0.97 – 0.34	**1.24** ***	0.68 – 1.80	0.11	-0.63 – 0.85	0.01	-0.39 – 0.42
	OS1	0.09	-0.56 – 0.74	0.33	-0.21 – 0.88	-0.26	-0.97 – 0.44	-0.28	-0.68 – 0.13
	OS2	**0.85** *	0.19 – 1.52	**1.31** ***	0.76 – 1.87	-0.14	-0.87 – 0.59	**0.70** ***	0.29 – 1.11
	OS3	**0.70** *	0.06 – 1.35	**1.68** ***	1.14 – 2.21	**-1.79** ***	-2.53 – -1.06	**0.49** *	0.09 – 0.89
	PR1	0.43	-0.23 – 1.09	**1.00** ***	0.46 – 1.54	**2.92** ***	2.05 – 3.79	**0.49** *	0.08 – 0.90
	PR2	**1.38** ***	0.72 – 2.03	**1.10** ***	0.56 – 1.64	**3.03** ***	2.16 – 3.91	**1.43** ***	1.02 – 1.83
	PR3	**1.17** ***	0.51 – 1.83	**1.32** ***	0.78 – 1.86	**3.09** ***	2.22 – 3.96	**1.38** ***	0.97 – 1.79
Habitats:Years	Inner slope: 2019	-0.85	-1.83 – 0.13	**-1.14** **	-1.96 – -0.32	-0.87	-2.00 – 0.26	-0.35	-0.95 – 0.26
	Inner slope: 2020	-0.24	-1.23 – 0.75	**-0.84** *	-1.67 – -0.01	-0.66	-1.79 – 0.48	**-0.85** **	-1.47 – -0.24
	Outer slope: 2019	0.07	-0.73 – 0.87	0.05	-0.61 – 0.70	0.45	-0.47 – 1.37	-0.07	-0.57 – 0.43
	Outer slope: 2020	-0.54	-1.35 – 0.26	-0.56	-1.22 – 0.10	-0.39	-1.31 – 0.53	**-0.75** **	-1.24 – -0.25

* *p<0.05* ** *p<0.01* *** *p<0.001*

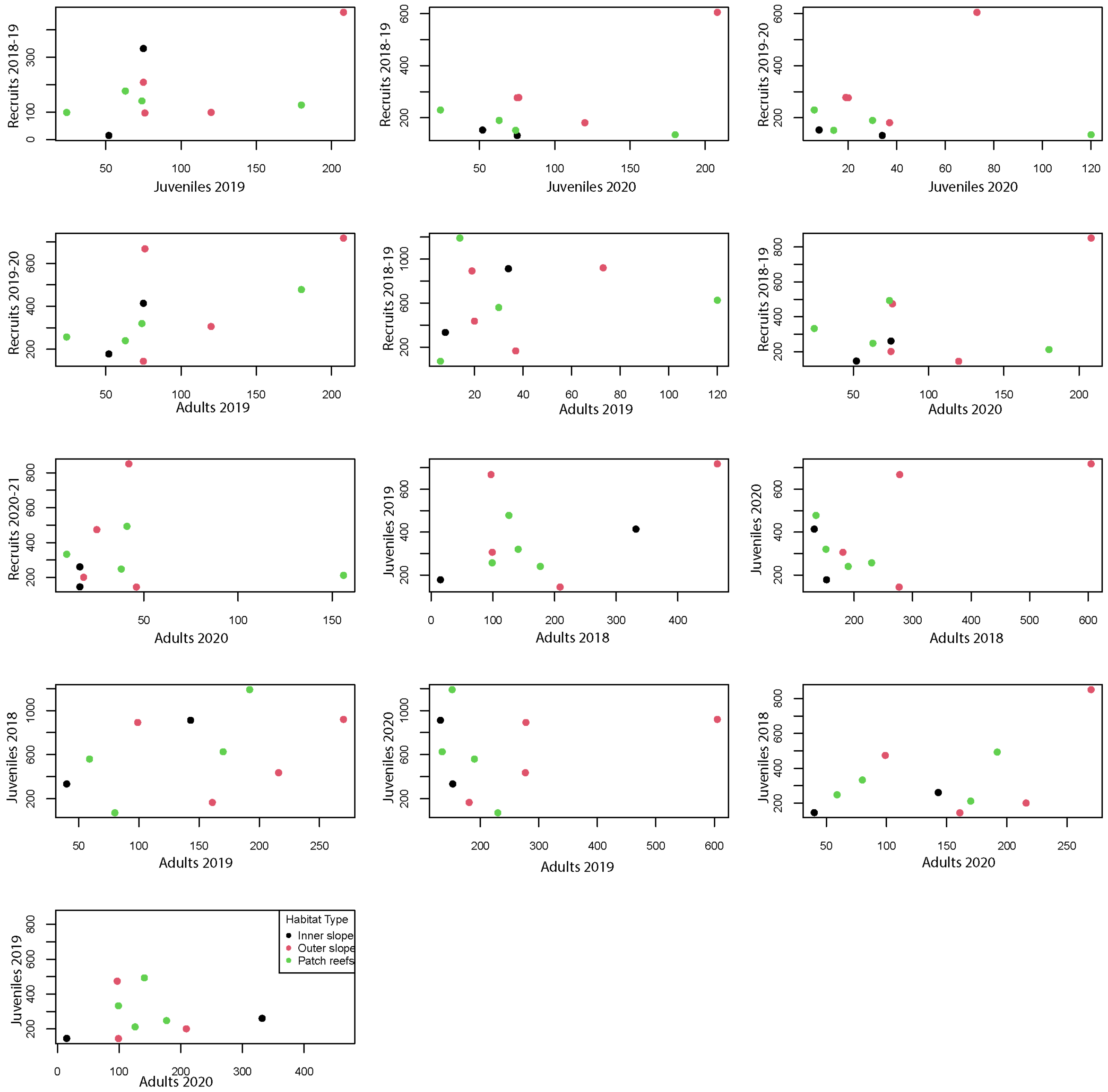

S3 Fig. Variation between abundance of recruit, juvenile and adult corals between the three years of the study for the overall assemblage (all taxa pooled).

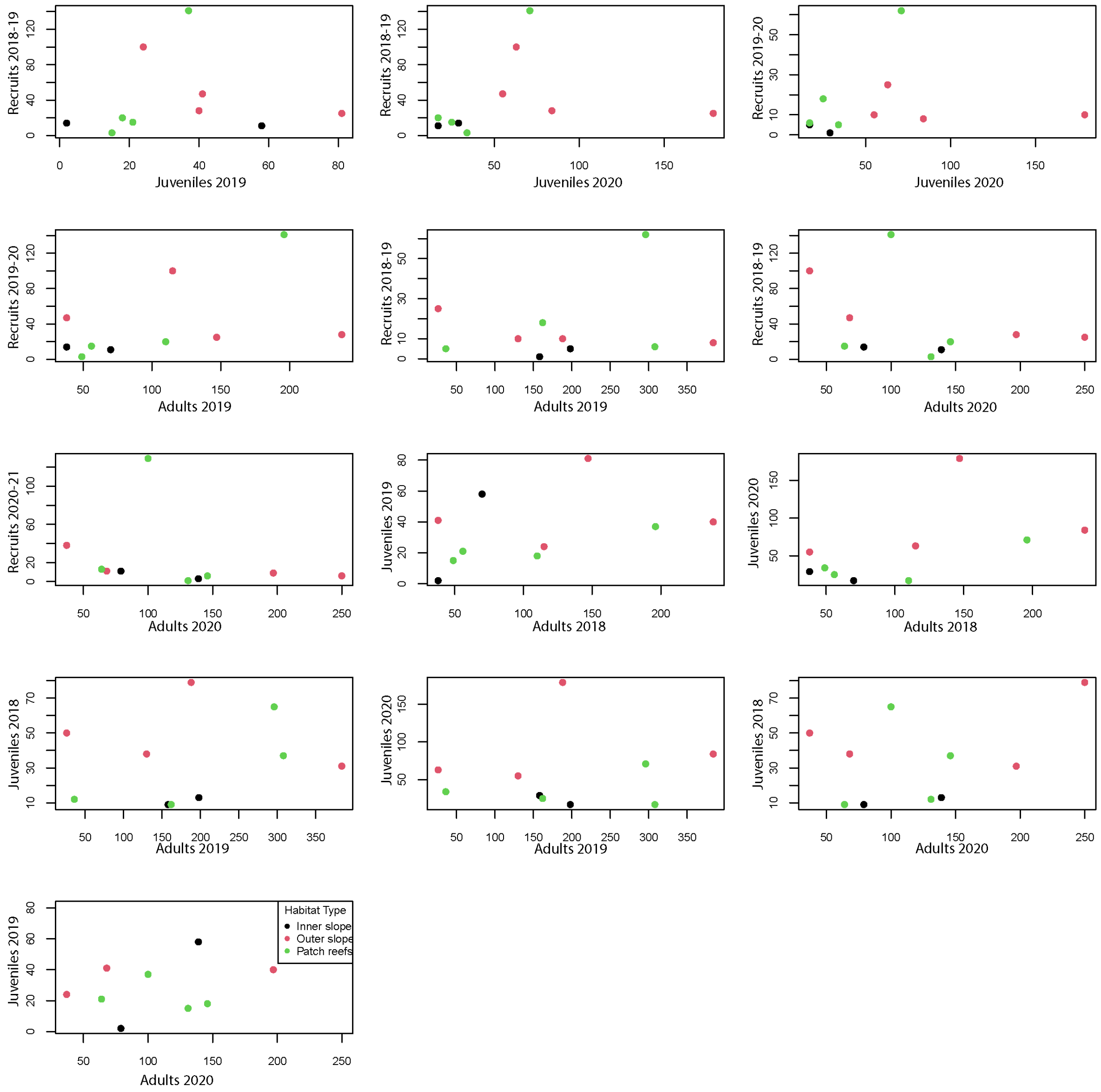

S4 Fig. Variation between abundance of recruit, juvenile and adult corals between the three years of the study for Acroporidae.

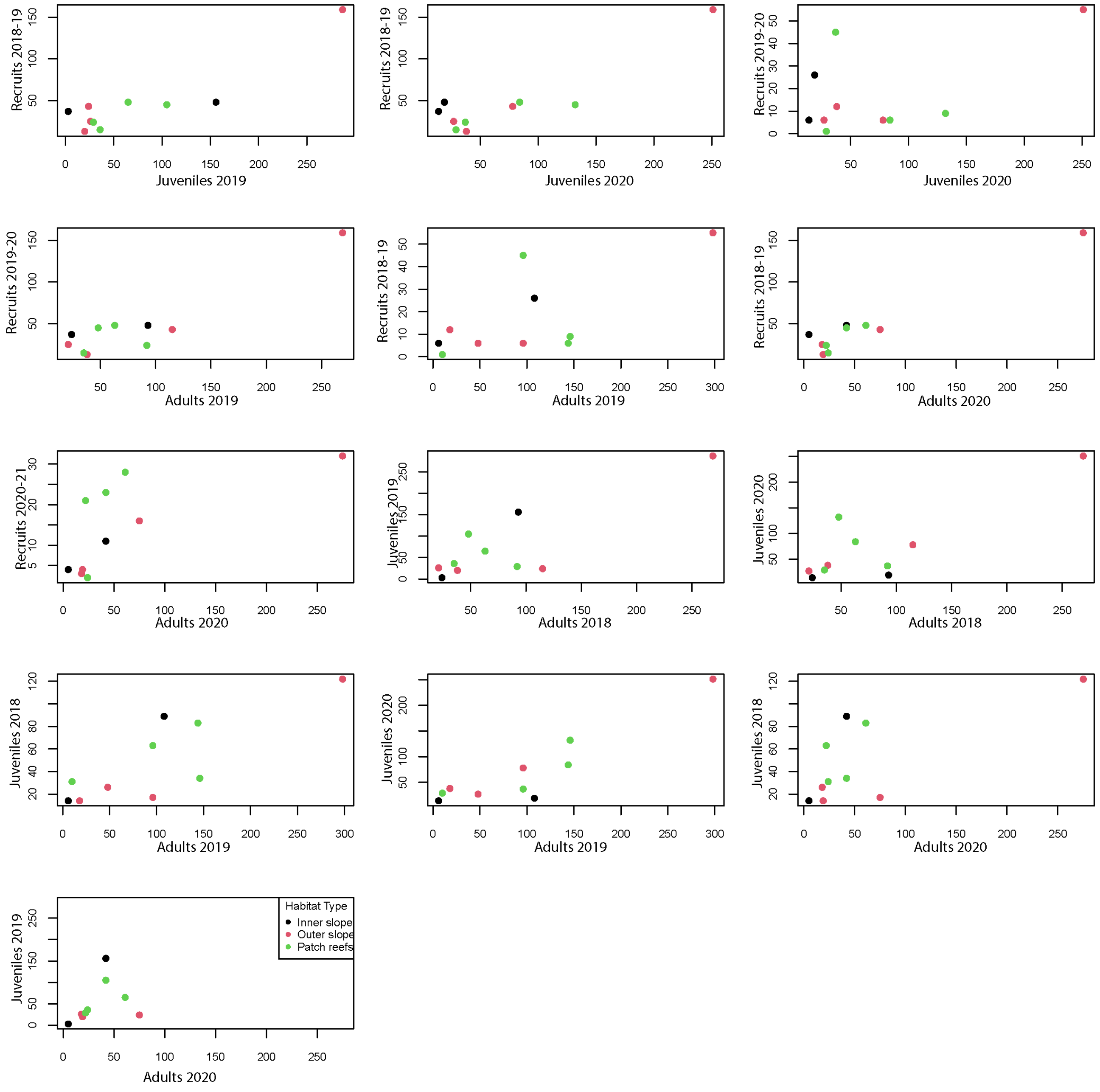

S5 Fig. Variation between abundance of recruit, juvenile and adult corals between the three years of the study for Pocilloporidae.

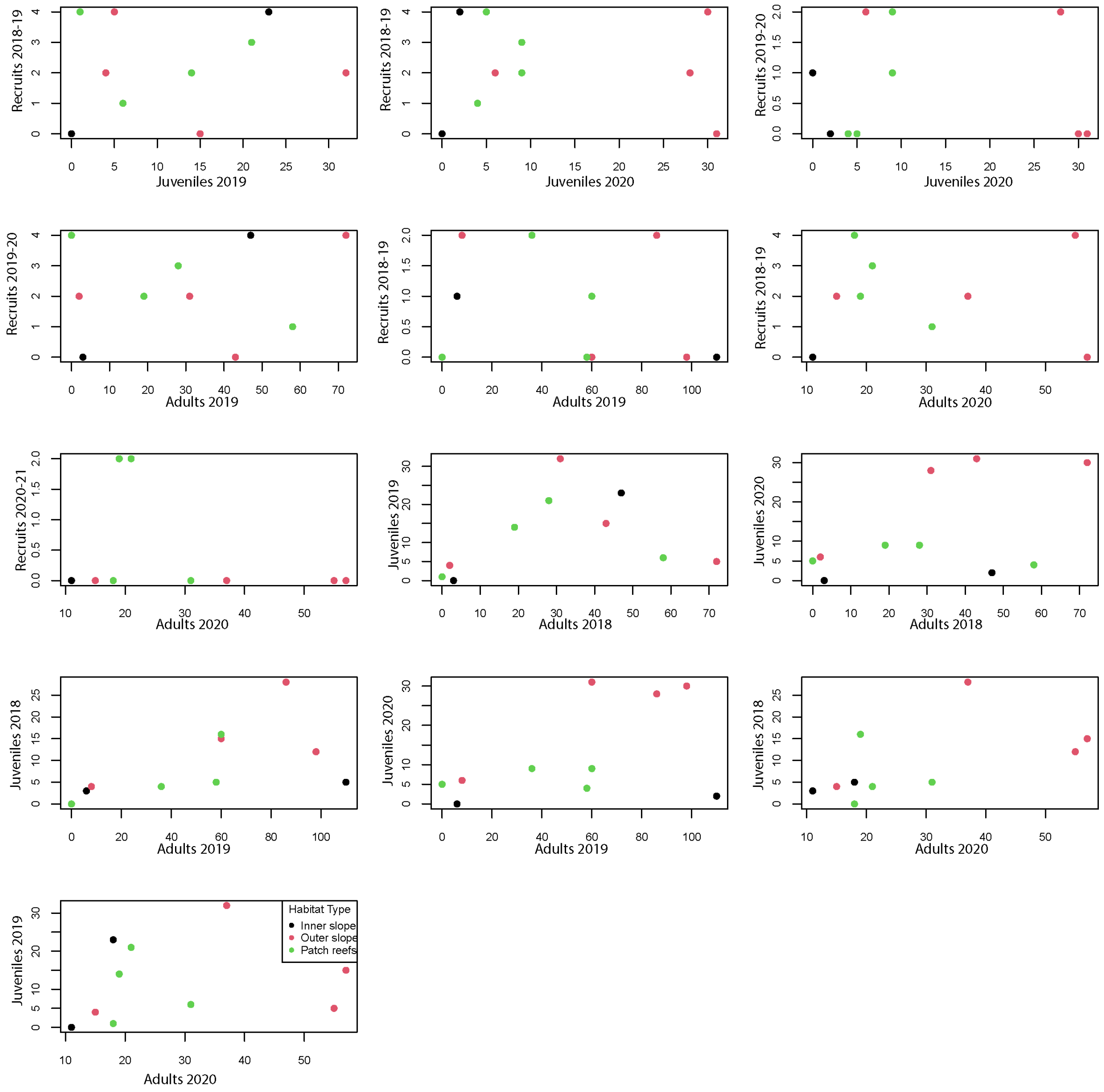

S6 Fig. Variation between abundance of recruit, juvenile and adult corals between the three years of the study for Poritidae.

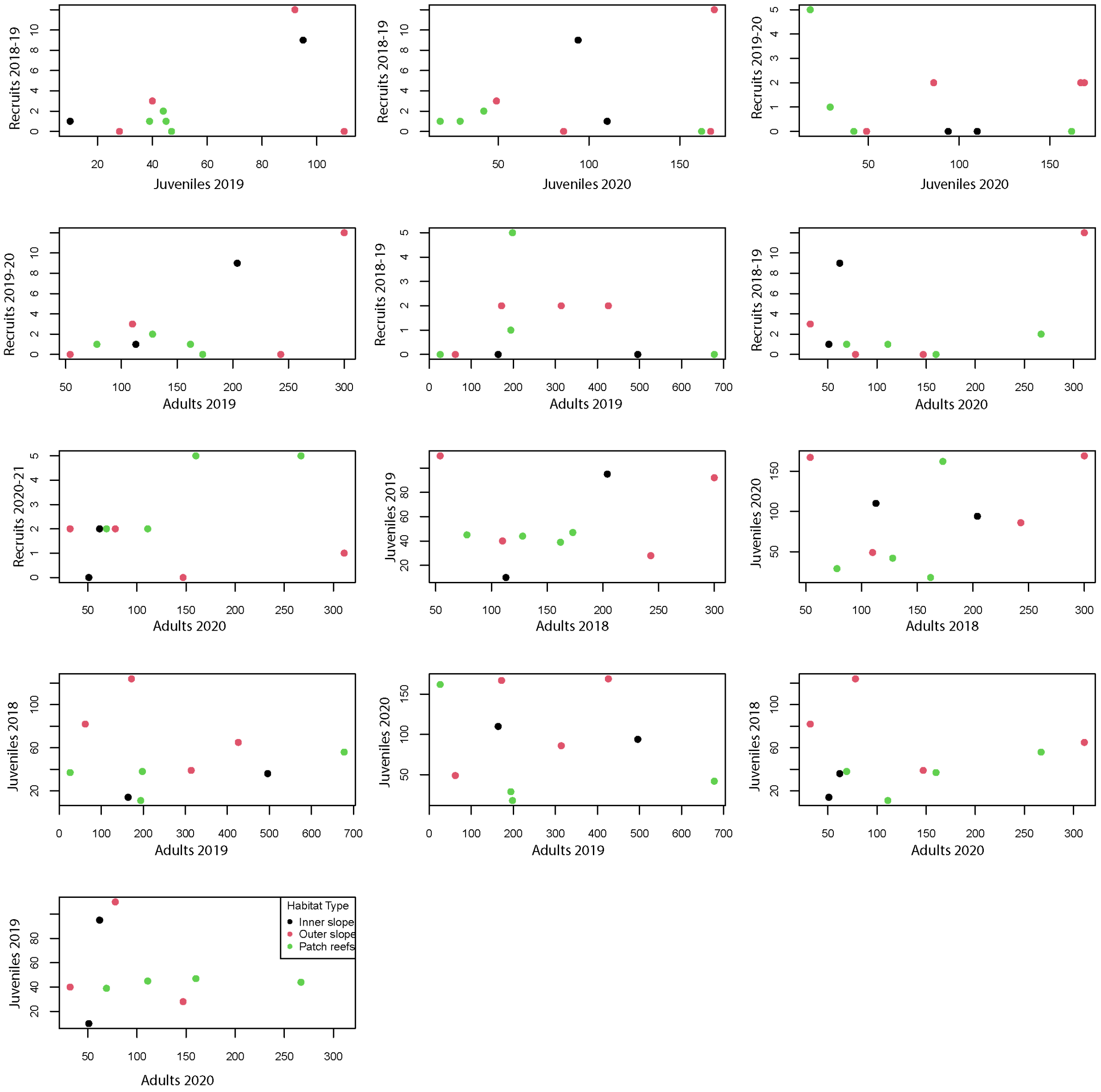

S7 Fig. Variation between abundance of recruit, juvenile and adult corals between the three years of the study for the category "other taxa".

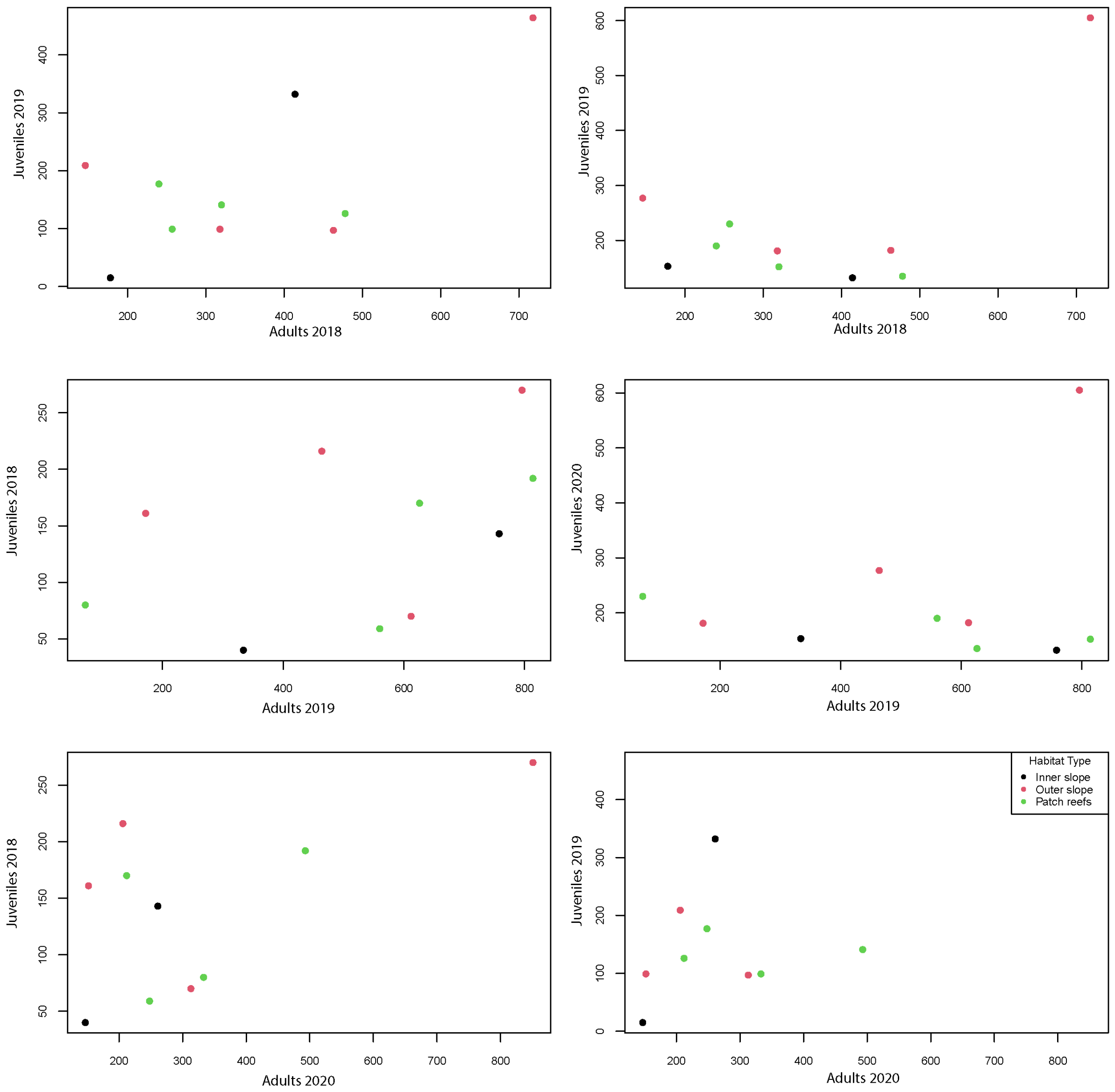

S8 Fig. Variation between abundance of juvenile and adult corals between the three years of the study for the overall assemblage (all genera pooled).

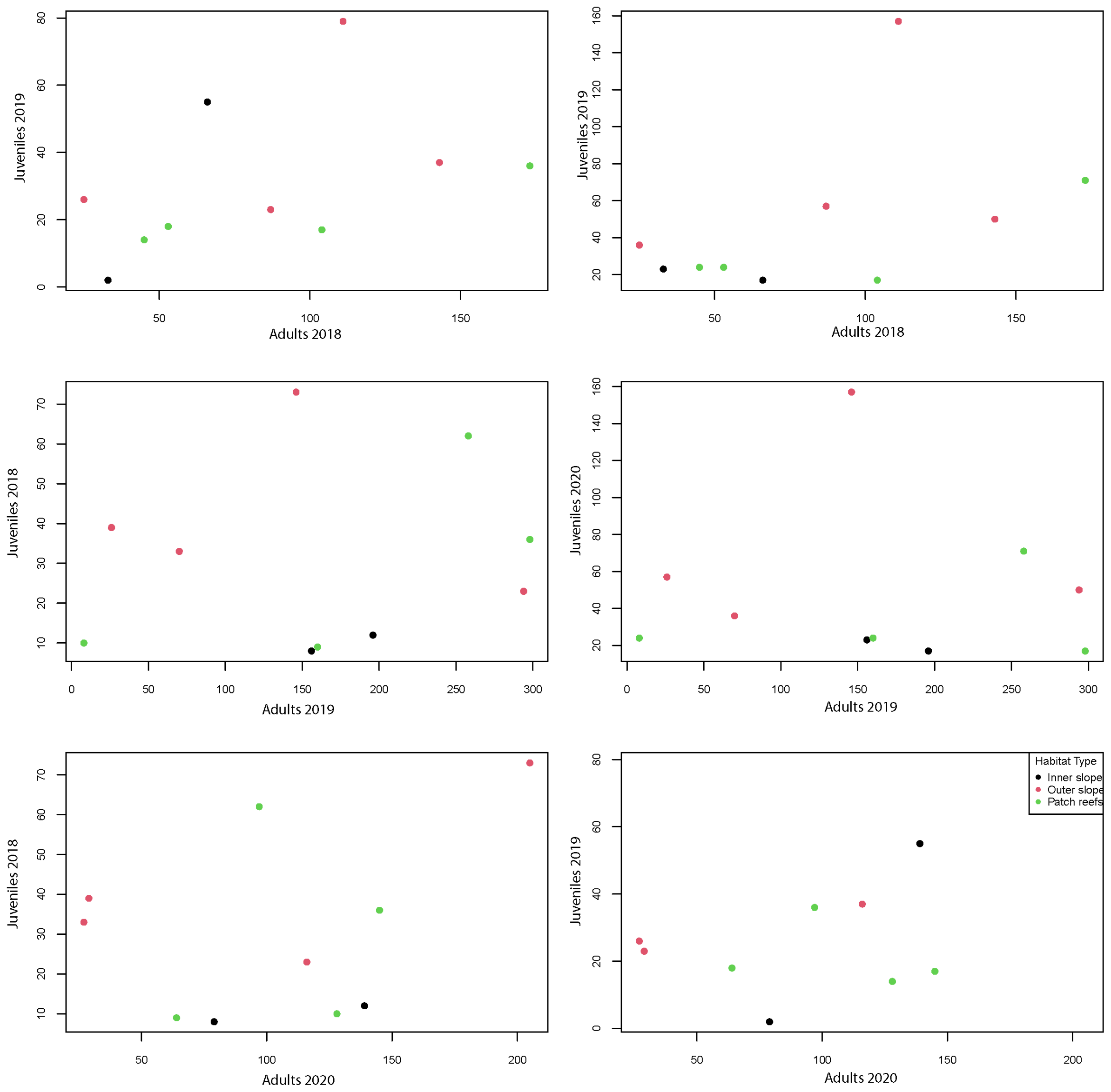

S9 Fig. Variation between abundance of juvenile and adult corals between the three years of the study for *Acropora*.

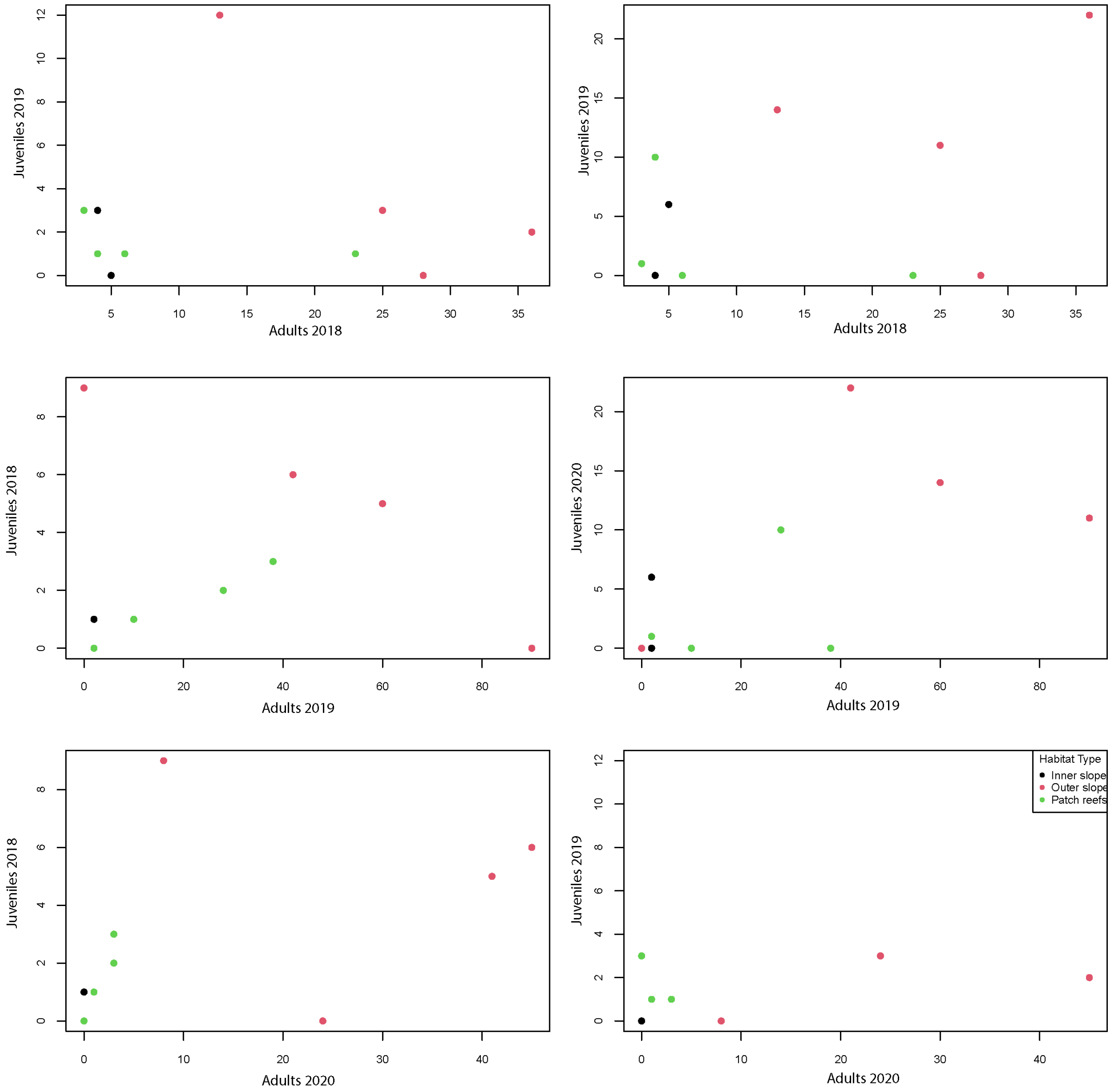

S10 Fig. Variation between abundance of juvenile and adult corals between the three years of the study for *Montipora*.

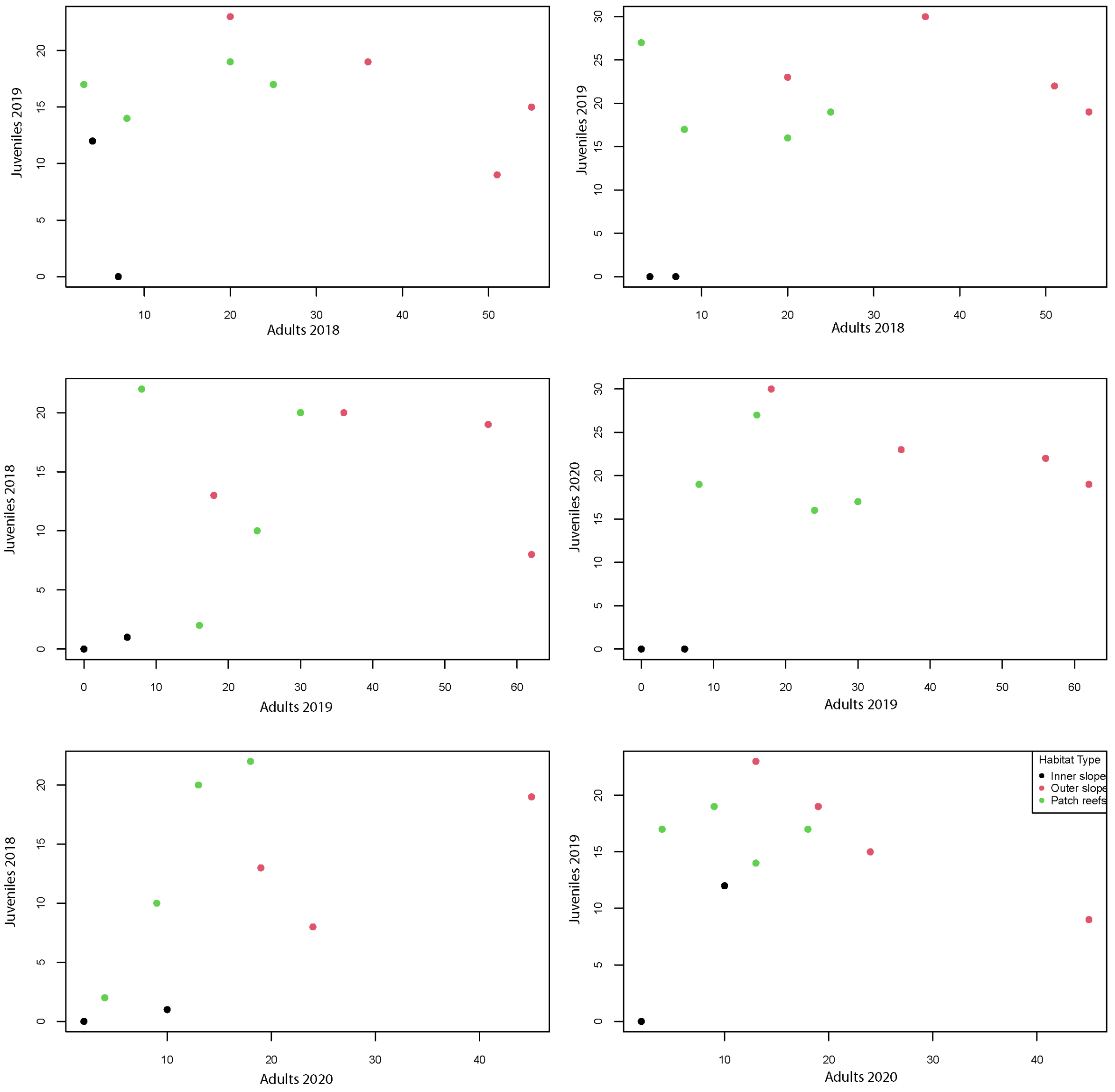

S11 Fig. Variation between abundance of juvenile and adult corals between the three years of the study for *Pocillopora*.

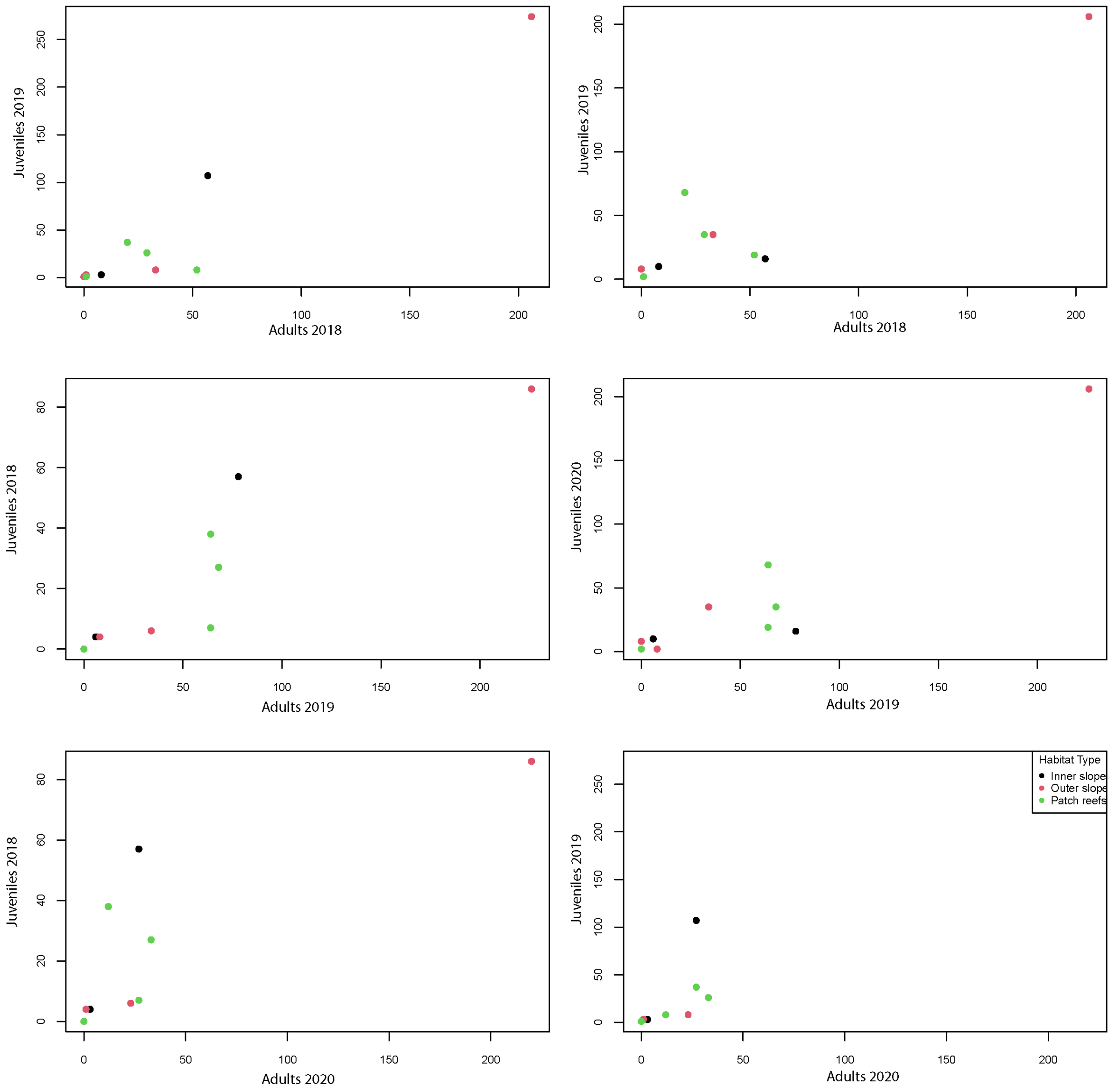

S12 Fig. Variation between abundance of juvenile and adult corals between the three years of the study for *Seriatopora*.

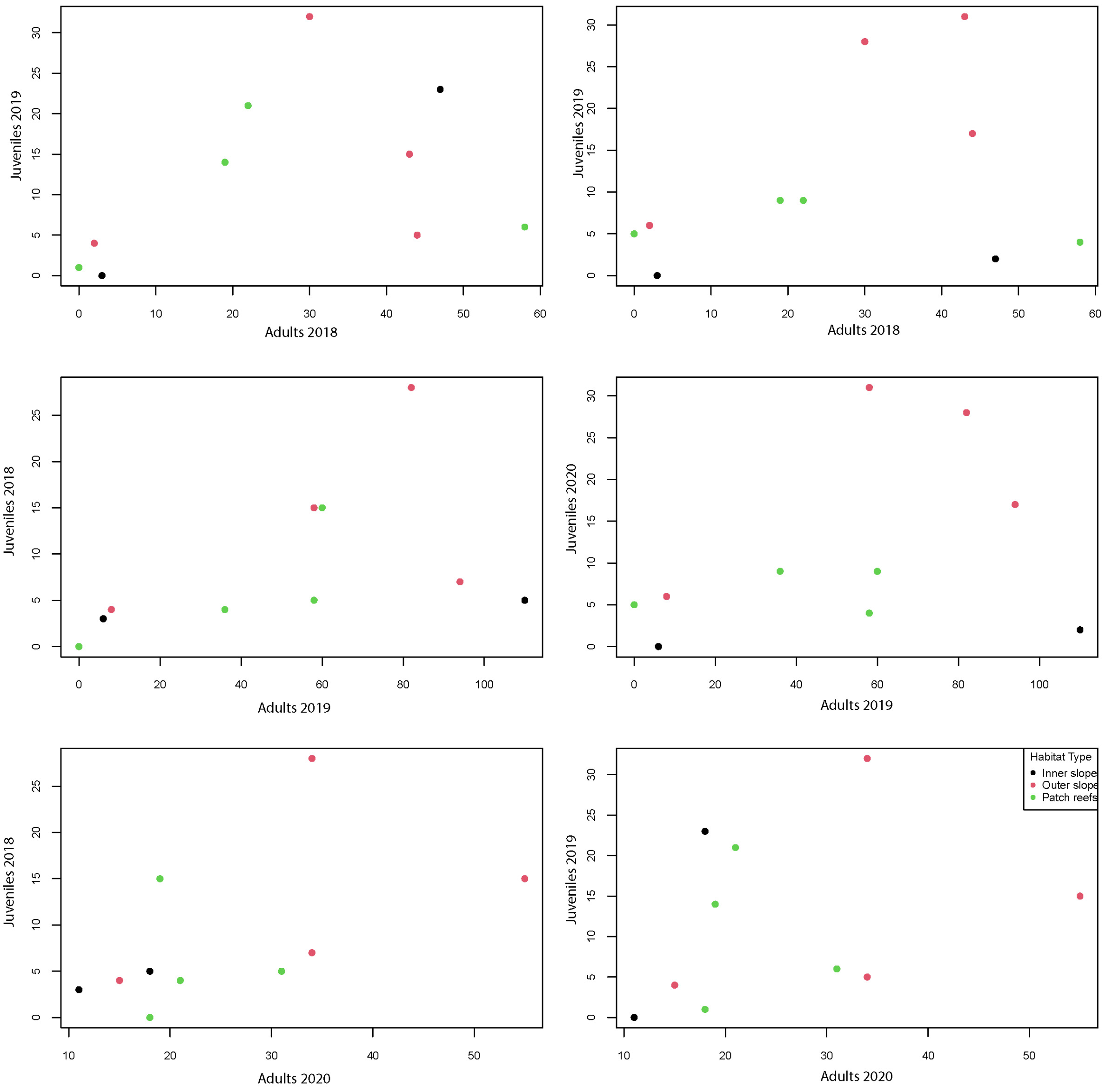

S13 Fig. Variation between abundance of juvenile and adult corals between the three years of the study for *Porites*.

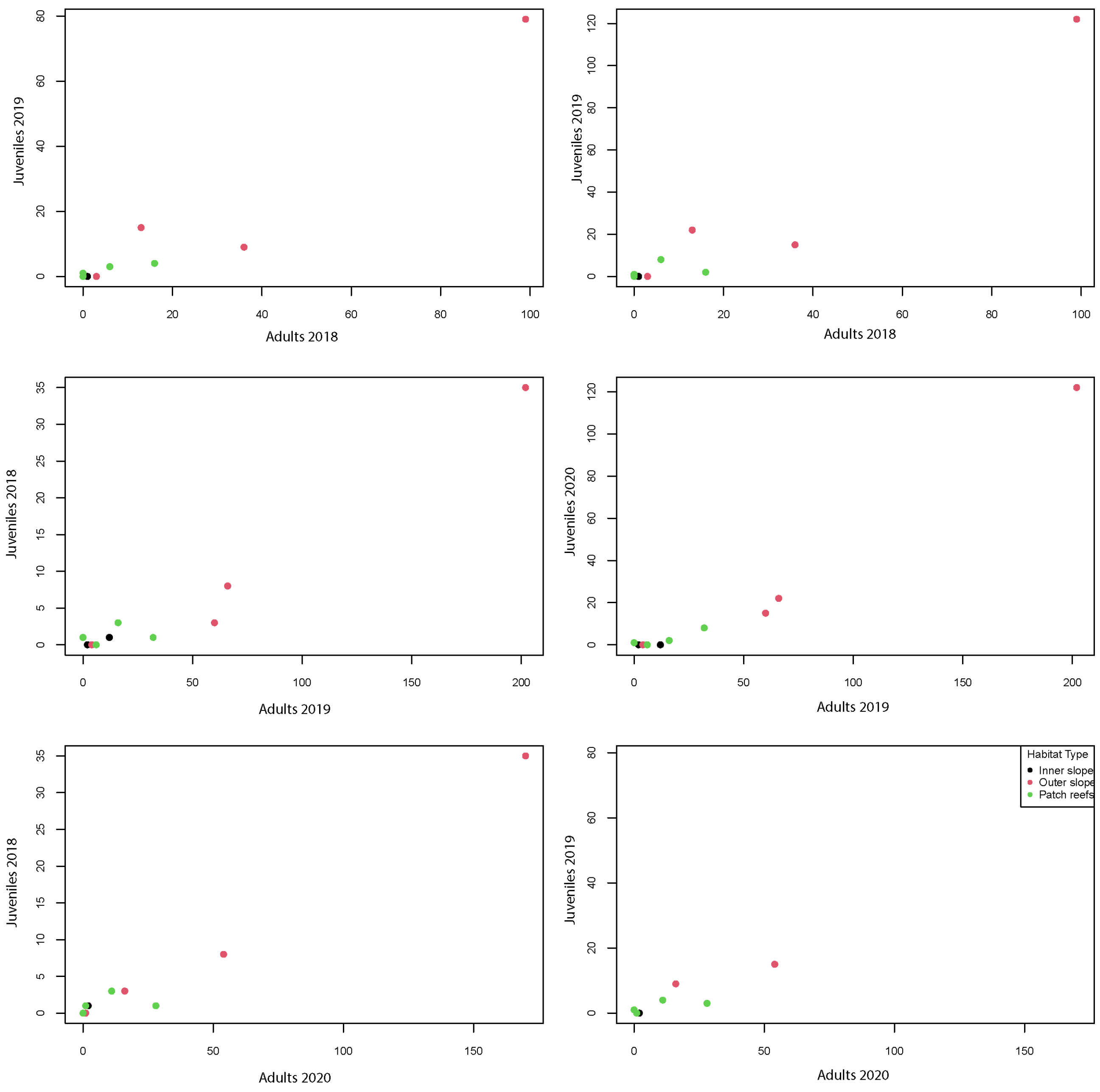

S14 Fig. Variation between abundance of juvenile and adult corals between the three years of the study for *Galaxea*.

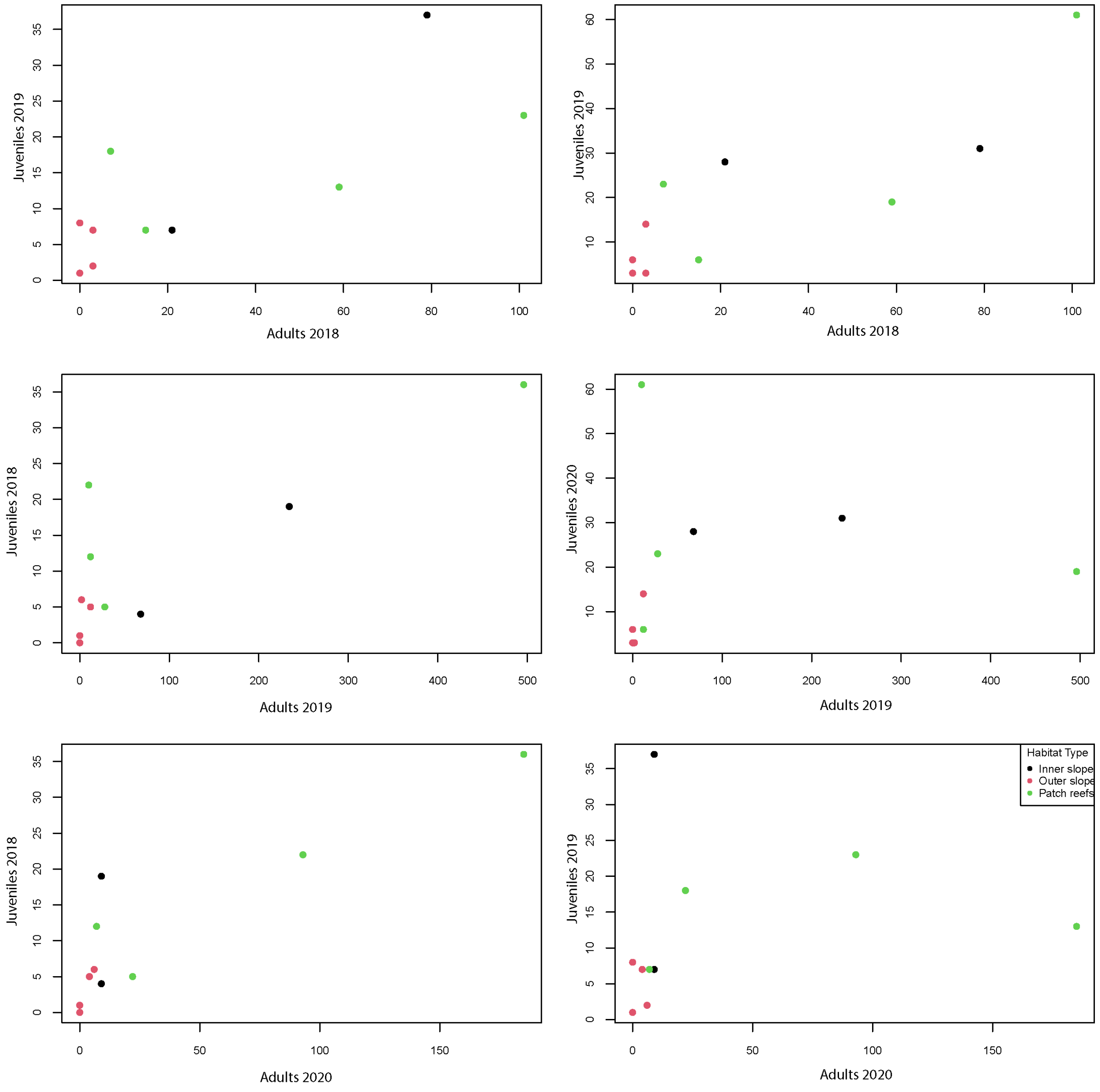

S15 Fig. Variation between abundance of juvenile and adult corals between the three years of the study for *Cycloseris*.

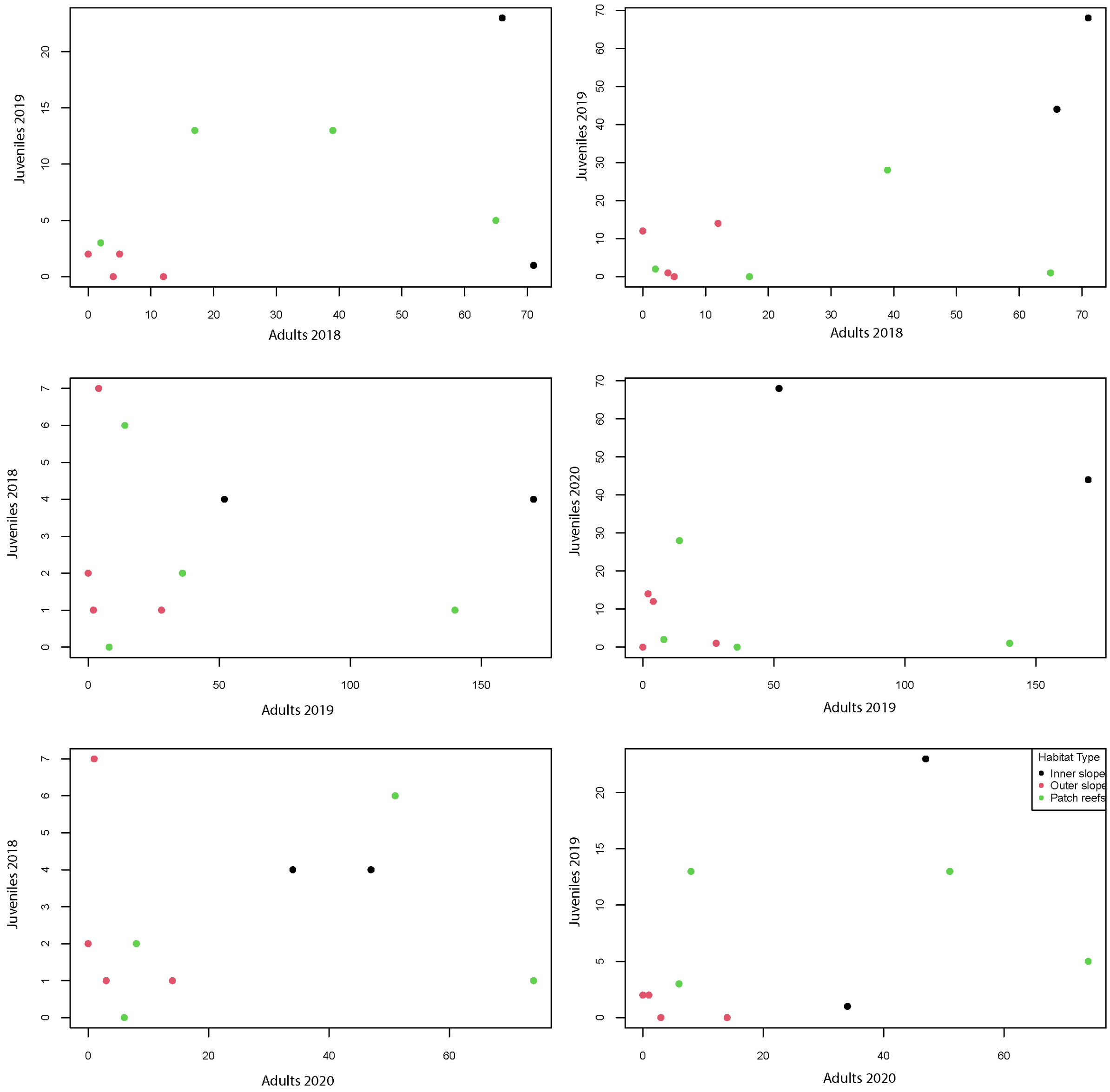

S16 Fig. Variation between abundance of juvenile and adult corals between the three years of the study for *Pavona*.
